# Reinforcement learning produces dominant strategies for the Iterated Prisoner’s Dilemma

**DOI:** 10.1371/journal.pone.0188046

**Published:** 2017-12-11

**Authors:** Marc Harper, Vincent Knight, Martin Jones, Georgios Koutsovoulos, Nikoleta E. Glynatsi, Owen Campbell

**Affiliations:** 1 Google Inc., Mountain View, CA, United States of America; 2 Cardiff University, School of Mathematics, Cardiff, United Kingdom; 3 Independent Researcher, Edinburgh, Scotland; 4 INRA, Université Côte d’Azur, CNRS, ISA, Nice, France; 5 Independent Researcher, Chester, United Kingdom; Southwest University, CHINA

## Abstract

We present tournament results and several powerful strategies for the Iterated Prisoner’s Dilemma created using reinforcement learning techniques (evolutionary and particle swarm algorithms). These strategies are trained to perform well against a corpus of over 170 distinct opponents, including many well-known and classic strategies. All the trained strategies win standard tournaments against the total collection of other opponents. The trained strategies and one particular human made designed strategy are the top performers in noisy tournaments also.

## Introduction

The Prisoner’s Dilemma (PD) is a two player game used to model a variety of strategic interactions. Each player chooses between cooperation (C) or defection (D). The payoffs of the game are defined by the matrix $\left(\begin{array}{cc} R & S\\ T & P \end{array}\right)$, where *T* > *R* > *P* > *S* and 2*R* > *T* + *S*. The PD is a one round game, but is commonly studied in a manner where the prior outcomes matter. This repeated form is called the Iterated Prisoner’s Dilemma (IPD). The IPD is frequently used to understand the evolution of cooperative behaviour from complex dynamics [1].

This manuscript uses the Axelrod library [2, 3], open source software for conducting IPD research with reproducibility as a principal goal. Written in the Python programming language, to date the library contains source code contributed by over 50 individuals from a variety of geographic locations and technical backgrounds. The library is supported by a comprehensive test suite that covers all the intended behaviors of all of the strategies in the library, as well as the features that conduct matches, tournaments, and population dynamics.

The library is continuously developed and as of version 3.0.0, the library contains over 200 strategies, many from the scientific literature, including classic strategies like Win Stay Lose Shift [4] and previous tournament winners such as OmegaTFT [5], Adaptive Pavlov [6], and ZDGTFT2 [7].

Since Robert Axelrod’s seminal tournament [8], a number of IPD tournaments have been undertaken and are summarised in Table 1. Further to the work described in [2] a regular set of standard, noisy [9] and probabilistic ending [10] tournaments are carried out as more strategies are added to the Axelrod library. Details and results are available here: http://axelrod-tournament.readthedocs.io. This work presents a detailed analysis of tournaments with 176 strategies.

**Table 1:** An overview of a selection of published tournaments. Not all tournaments were ‘standard’ round robins; for more details see the indicated references.

Year	Reference	Number of Strategies	Type	Source Code
1979	[8]	13	Standard	Not immediately available
1979	[10]	64	Standard	Available in FORTRAN
1991	[9]	13	Noisy	Not immediately available
2002	[11]	16	Wildlife	Not applicable
2005	[12]	223	Varied	Not available
2012	[7]	13	Standard	Not fully available
2016	[2]	129	Standard	Fully available

In this work we describe how collections of strategies in the Axelrod library have been used to train new strategies specifically to win IPD tournaments. These strategies are trained using generic strategy archetypes based on e.g. finite state machines, arriving at particularly effective parameter choices through evolutionary or particle swarm algorithms. There are several previous publications that use evolutionary algorithms to evolve IPD strategies in various circumstances [13–22]. See also [23] for a strategy trained to win against a collection of well-known IPD opponents and see [24] for a prior use of particle swarm algorithms. Our results are unique in that we are able to train against a large and diverse collection of strategies available from the scientific literature. Crucially, the software used in this work is openly available and can be used to train strategies in the future in a reliable manner, with confidence that the opponent strategies are correctly implemented, tested and documented.

## Materials and methods

### The strategy archetypes

The Axelrod library now contains many parametrised strategies trained using machine learning methods. Most are deterministic, use many rounds of memory, and perform extremely well in tournaments as will be discussed in the results Section. Training will be discussed in a later section. These strategies can encode a variety of other strategies, including classic strategies like Tit For Tat [25], handshake strategies, and grudging strategies, that always defect after an opponent defection.

#### LookerUp

**Fig 1:**
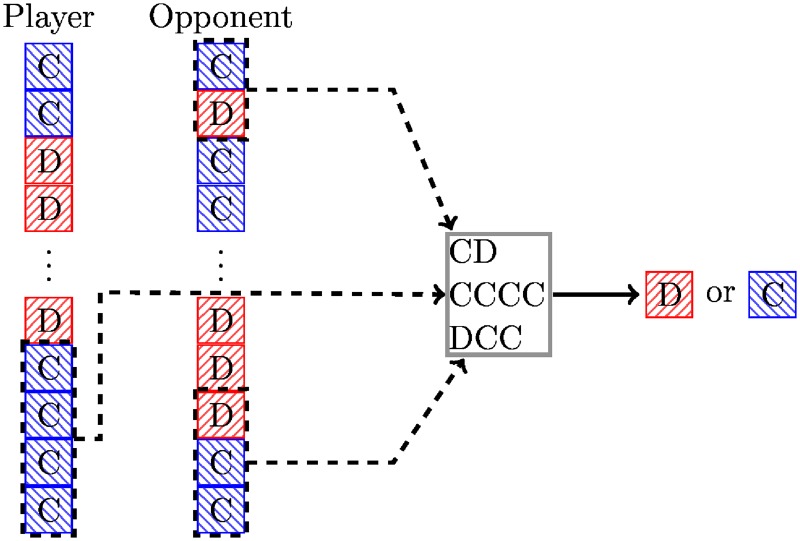
Diagrammatic representation of the looker up archetype.

The LookerUp strategy is based on a lookup table and encodes a set of deterministic responses based on the opponent’s first *n*_1_ moves, the opponent’s last *m*_1_ moves, and the players last *m*_2_ moves. If *n*_1_ > 0 then the player has infinite memory depth, otherwise it has depth max(*m*_1_, *m*_2_). This is illustrated diagrammatically in Fig 1.

Training of this strategy corresponds to finding maps from partial histories to actions, either a cooperation or a defection. Although various combinations of *n*_1_, *m*_1_, and *m*_2_ have been tried, the best performance at the time of training was obtained for *n*_1_ = *m*_1_ = *m*_2_ = 2 and generally for *n*_1_ > 0. A strategy called EvolvedLookerUp2_2_2 is among the top strategies in the library.

This archetype can be used to train deterministic memory-*n* strategies with the parameters *n*_1_ = 0 and *m*_1_ = *m*_2_ = *n*. For *n* = 1, the resulting strategy cooperates if the last round was mutual cooperation and defects otherwise, known as Grim or Grudger.

Two strategies in the library, Winner12 and Winner21, from [26], are based on lookup tables for *n*_1_ = 0, *m*_1_ = 1, and *m*_2_ = 2. The strategy Winner12 emerged in less than 10 generations of training in our framework using a score maximizing objective. Strategies nearly identical to Winner21 arise from training with a Moran process objective.

#### Gambler

**Fig 2:**
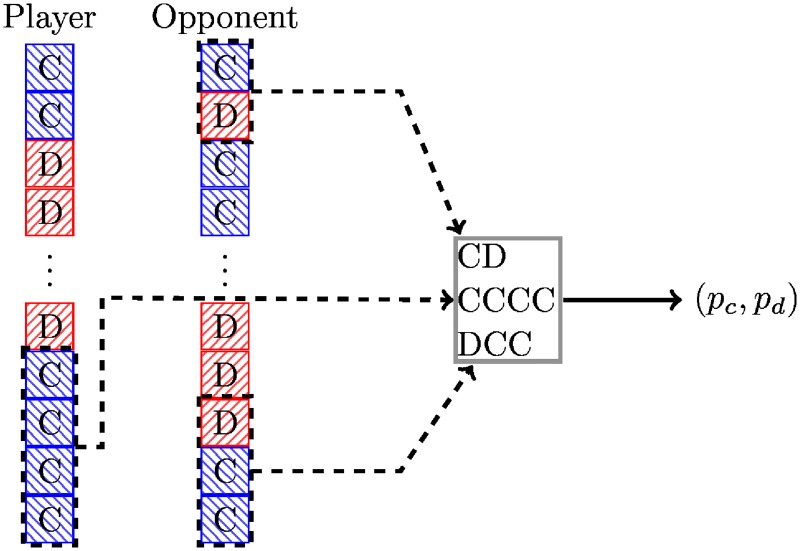
Diagrammatic representation of the Gambler archetype.

Gambler is a stochastic variant of LookerUp. Instead of deterministically encoded moves the lookup table emits probabilities which are used to choose cooperation or defection. This is illustrated diagrammatically in Fig 2.

Training of this strategy corresponds to finding maps from histories to a probability of cooperation. The library includes a strategy trained with *n*_1_ = *m*_1_ = *m*_2_ = 2 that is *mostly deterministic*, with 52 of the 64 probabilities being 0 or 1.

This strategy type can be used to train arbitrary memory-*n* strategies. A memory one strategy called PSOGamblerMem1 was trained, with probabilities (Pr(C | CC), Pr(C | CD), Pr(C | DC), Pr(C | DD)) = (1, 0.5217, 0, 0.121). Though it performs well in standard tournaments (see Table 2) it does not outperform the longer memory strategies, and is bested by a similar strategy that also uses the first round of play: PSOGambler_1_1_1.

**Table 2:** Standard tournament: Mean score per turn of top 15 strategies (ranked by median over 50000 tournaments). The leaderboard is dominated by the trained strategies (indicated by a *).

	mean	std	min	5%	25%	50%	75%	95%	max
EvolvedLookerUp2_2_2*	2.955	0.010	2.915	2.937	2.948	2.956	2.963	2.971	2.989
Evolved HMM 5*	2.954	0.014	2.903	2.931	2.945	2.954	2.964	2.977	3.007
Evolved FSM 16*	2.952	0.013	2.900	2.930	2.943	2.953	2.962	2.973	2.993
PSO Gambler 2_2_2*	2.938	0.013	2.884	2.914	2.930	2.940	2.948	2.957	2.972
Evolved FSM 16 Noise 05*	2.919	0.013	2.874	2.898	2.910	2.919	2.928	2.939	2.965
PSO Gambler 1_1_1*	2.912	0.023	2.805	2.874	2.896	2.912	2.928	2.950	3.012
Evolved ANN 5*	2.912	0.010	2.871	2.894	2.905	2.912	2.919	2.928	2.945
Evolved FSM 4*	2.910	0.012	2.867	2.889	2.901	2.910	2.918	2.929	2.943
Evolved ANN*	2.907	0.010	2.865	2.890	2.900	2.908	2.914	2.923	2.942
PSO Gambler Mem1*	2.901	0.025	2.783	2.858	2.884	2.901	2.919	2.942	2.994
Evolved ANN 5 Noise 05*	2.864	0.008	2.830	2.850	2.858	2.865	2.870	2.877	2.891
DBS	2.857	0.009	2.823	2.842	2.851	2.857	2.863	2.872	2.899
Winner12	2.849	0.008	2.820	2.836	2.844	2.850	2.855	2.862	2.874
Fool Me Once	2.844	0.008	2.818	2.830	2.838	2.844	2.850	2.857	2.882
Omega TFT: 3, 8	2.841	0.011	2.800	2.822	2.833	2.841	2.849	2.859	2.882

These strategies are trained with a particle swarm algorithm rather than an evolutionary algorithm (though the former would suffice). Particle swarm algorithms have been used to trained IPD strategies previously [24].

#### ANN: Single hidden layer artificial neural network

Strategies based on artificial neural networks use a variety of features computed from the history of play:

- Opponent’s first move is C
- Opponent’s first move is D
- Opponent’s second move is C
- Opponent’s second move is D
- Player’s previous move is C
- Player’s previous move is D
- Player’s second previous move is C
- Player’s second previous move is D
- Opponent’s previous move is C
- Opponent’s previous move is D
- Opponent’s second previous move is C
- Opponent’s second previous move is D
- Total opponent cooperations
- Total opponent defections
- Total player cooperations
- Total player defections
- Round number

**Fig 3:**
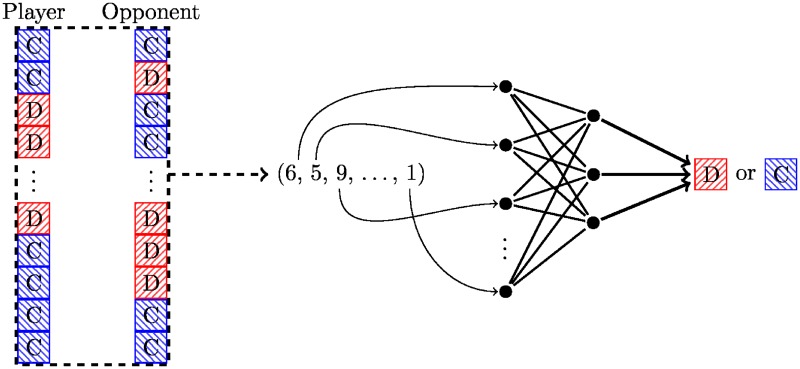
Diagrammatic representation of the ANN archetype.

These are then input into a feed forward neural network with one layer and user-supplied width. This is illustrated diagrammatically in Fig 3.

Training of this strategy corresponds to finding parameters of the neural network. An inner layer with just five nodes performs quite well in both deterministic and noisy tournaments. The output of the ANN used in this work is deterministic; a stochastic variant that outputs probabilities rather than exact moves could be created.

#### Finite state machines

**Fig 4:**
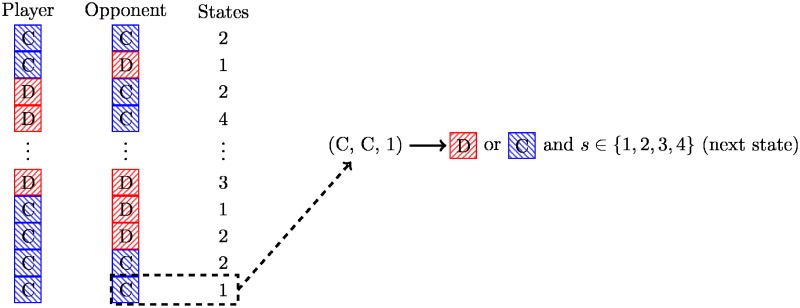
Diagrammatic representation of the finite state machine archetype.

Strategies based on finite state machines are deterministic and computationally efficient. In each round of play the strategy selects an action based on the current state and the opponent’s last action, transitioning to a new state for the next round. This is illustrated diagrammatically in Fig 4.

**Fig 5:**
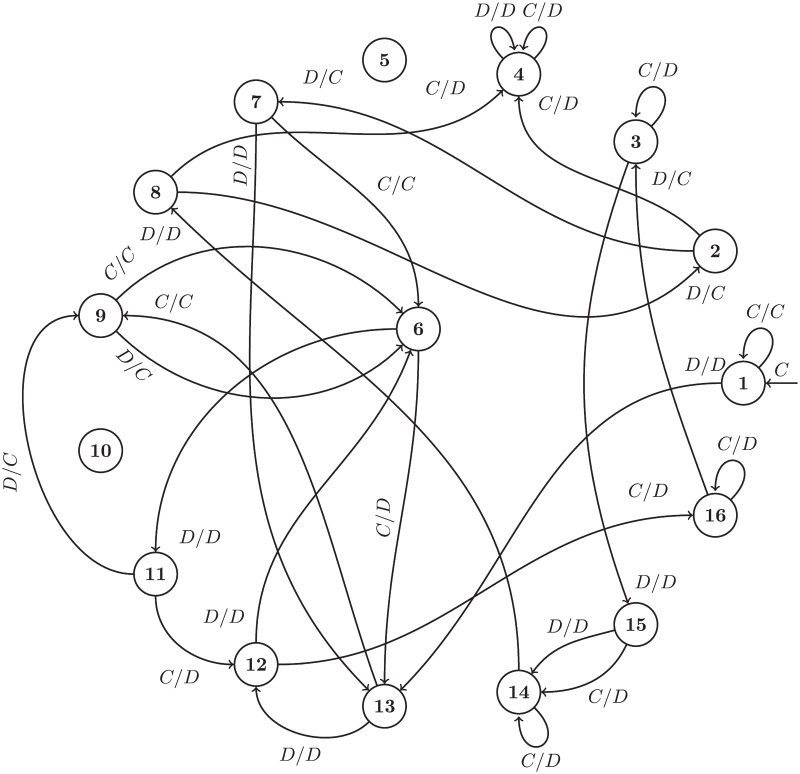
Evolved_FSM_16: Trained to maximize score in a standard tournament.

**Fig 6:**
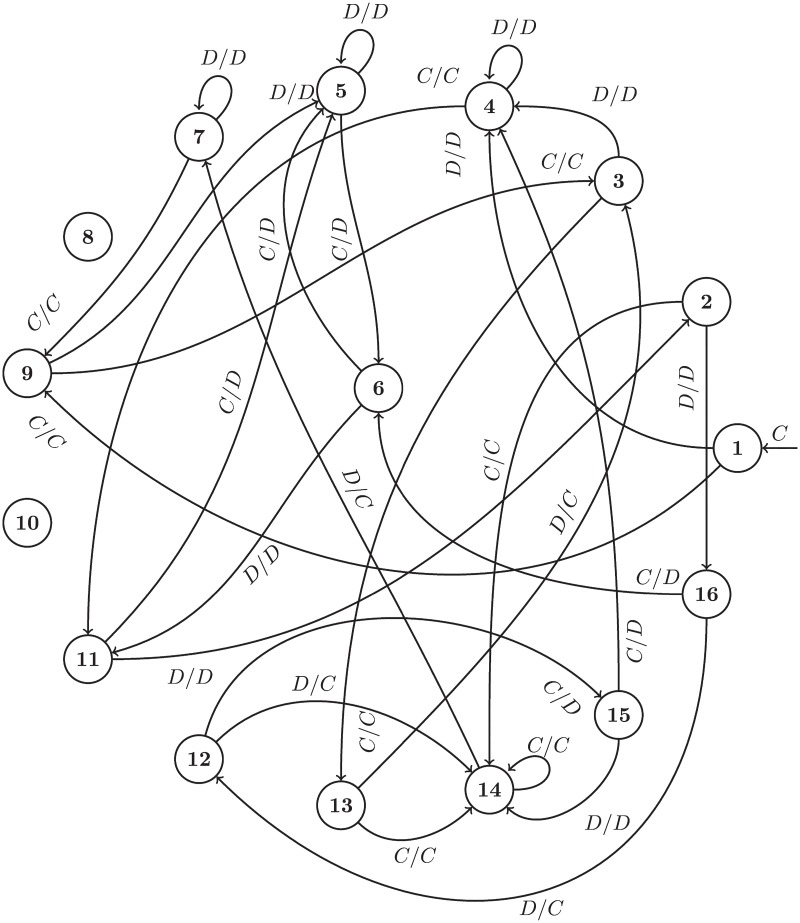
Evolved_FSM_16_Noise_05: Trained to maximize score in a noisy tournament.

Training this strategy corresponds to finding mappings of states and histories to an action and a state. Figs 5 and 6 show two of the trained finite state machines. The layout of state nodes is kept the same between Figs 5 and 6 to highlight the effect of different training environments. Note also that two of the 16 states are not used, this is also an outcome of the training process.

#### Hidden markov models

**Fig 7:**
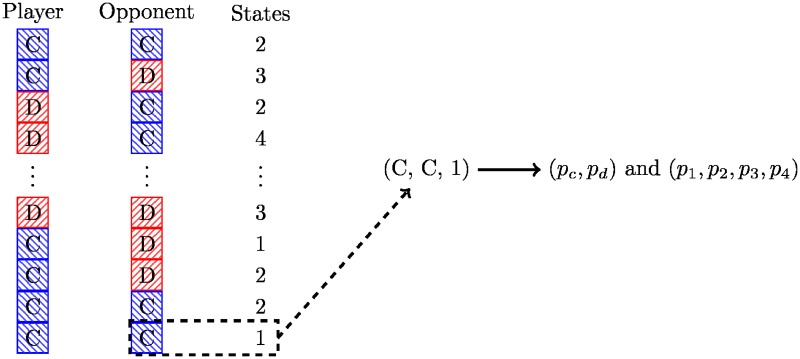
Diagrammatic representation of the hidden markov model archetype.

A variant of finite state machine strategies are called hidden Markov models (HMMs). Like the strategies based on finite state machines, these strategies also encode an internal state. However, they use probabilistic transitions based on the prior round of play to other states and cooperate or defect with various probabilities at each state. This is shown diagrammatically in Fig 7. Training this strategy corresponds to finding mappings of states and histories to probabilities of cooperating as well as probabilities of the next internal state.

#### Meta strategies

There are several strategies based on ensemble methods that are common in machine learning called Meta strategies. These strategies are composed of a team of other strategies. In each round, each member of the team is polled for its desired next move. The ensemble then selects the next move based on a rule, such as the consensus vote in the case of MetaMajority or the best individual performance in the case of MetaWinner. These strategies were among the highest performing in the library before the inclusion of those trained by reinforcement learning.

Because these strategies inherit many of the properties of the strategies on which they are based, including using knowledge of the match length to defect on the last round(s) of play, not all of these strategies were included in results of this paper. These strategies do not typically outperform the trained strategies described above.

### Training methods

The trained strategies (denoted by a * in Appendix A) were trained using reinforcement learning algorithms. The ideas of reinforcement learning can be attributed to the original work of [27] in which the notion that computers would learn by taking random actions but according to a distribution that picked actions with high rewards more often. The two particular algorithms used here:

- Particle Swarm Algorithm: [28].
- Evolutionary algorithm: [29].

The Particle Swarm Algorithm is implemented using the pyswarm library: https://pypi.python.org/pypi/pyswarm. This algorithm was used only to train the Gambler archetype.

All other strategies were trained using evolutionary algorithms. The evolutionary algorithms used standard techniques, varying strategies by mutation and crossover, and evaluating the performance against each opponent for many repetitions. The best performing strategies in each generation are persisted, variants created, and objective functions computed again.

The default parameters for this procedure:

- A population size of 40 individuals (kept constant across the generations);
- A mutation rate of 10%;
- 10 individuals kept from one generation to the next;
- A total of 500 generations.

All implementations of these algorithms are archived at [30]. This software is (similarly to the Axelrod library) available on github https://github.com/Axelrod-Python/axelrod-dojo. There are objective functions for:

- total or mean payoff,
- total or mean payoff difference (unused in this work),
- total Moran process wins (fixation probability). This lead to the strategies named TF1, TF2, TF3 listed in Appendix A.

These can be used in noisy or standard environments. These objectives can be further modified to suit other purposes. New strategies could be trained with variations including spatial structure and probabilistically ending matches.

## Results

This section presents the results of a large IPD tournament with strategies from the Axelrod library, including some additional parametrized strategies (e.g. various parameter choices for Generous Tit For Tat [23]). These are listed in Appendix A.

All strategies in the tournament follow a simple set of rules in accordance with earlier tournaments:

- Players are unaware of the number of turns in a match.
- Players carry no acquired state between matches.
- Players cannot observe the outcome of other matches.
- Players cannot identify their opponent by any label or identifier.
- Players cannot manipulate or inspect their opponents in any way.

Any strategy that does not follow these rules, such as a strategy that defects on the last round of play, was omitted from the tournament presented here (but not necessarily from the training pool).

**Fig 8:**
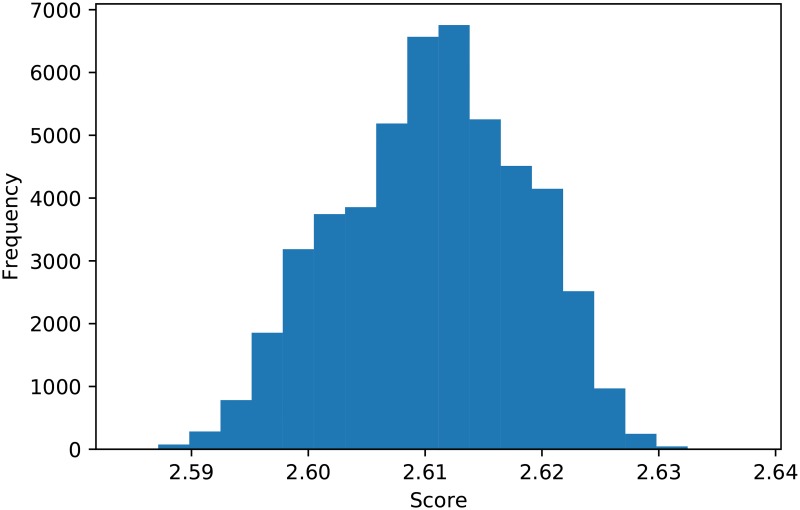
Scores for Tit for Tat over 50000 tournaments.

**Fig 9:**
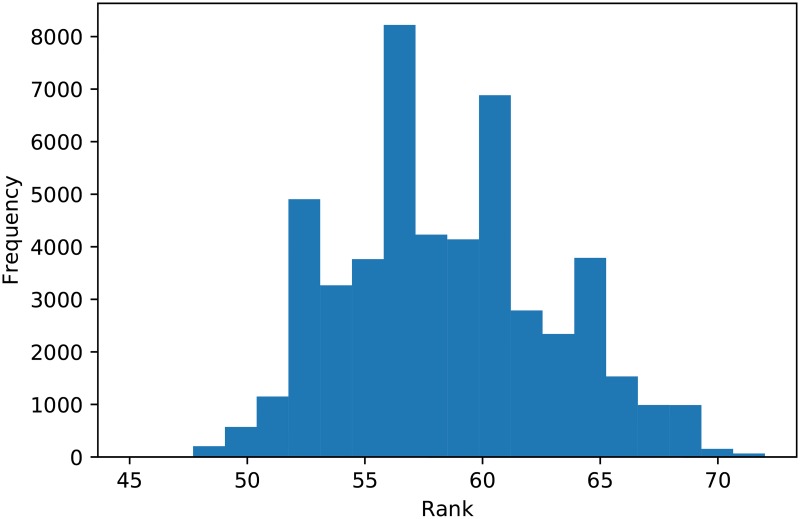
Ranks for Tit for Tat over 50000 tournaments.

**Fig 10:**
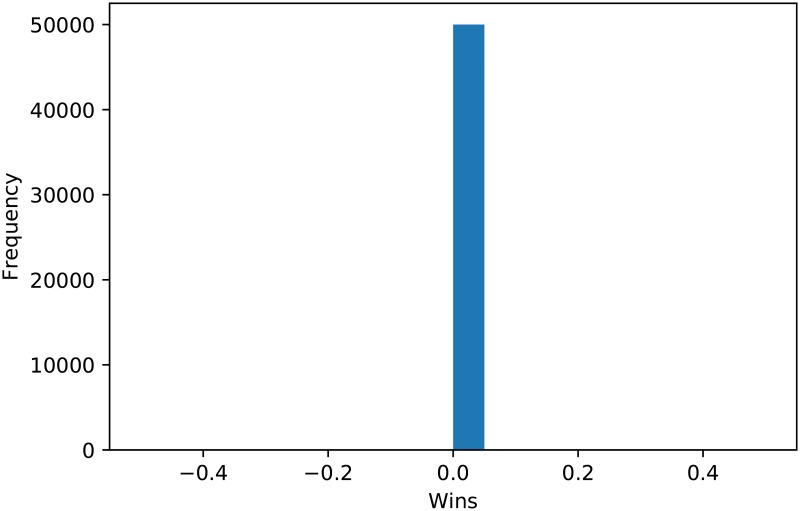
Wins for Tit for Tat over 50000 tournaments.

A total of 176 are included, of which 53 are stochastic. A standard tournament with 200 turns and a tournament with 5% noise is discussed. Due to the inherent stochasticity of these IPD tournaments, these tournaments were repeated 50000 times. This allows for a detailed and confident analysis of the performance of strategies. To illustrate the results considered, Fig 8 shows the distribution of the mean score per turn of Tit For Tat over all the repetitions. Similarly, Fig 9 shows the ranks of of Tit For Tat for each repetition. (We note that it never wins a tournament). Finally Fig 10 shows the number of opponents beaten in any given tournament: Tit For Tat does not win any match. (This is due to the fact that it will either draw with mutual cooperation or defect second).

The utilities used are (*R*, *P*, *T*, *S*) = (3, 1, 5, 0) thus the specific Prisoner’s Dilemma being played is:

$$\left(\begin{array}{cc} 3 & 0\\ 5 & 1 \end{array}\right)$$ (1)

All data generated for this work is archived and available at [31].

### Standard tournament

The top 11 performing strategies by median payoff are all strategies trained to maximize total payoff against a subset of the strategies (Table 2). The next strategy is Desired Belief Strategy (DBS) [32], which actively analyzes the opponent and responds accordingly. The next two strategies are Winner12, based on a lookup table, Fool Me Once [3], a grudging strategy that defects indefinitely on the second defection, and Omega Tit For Tat [12].

**Fig 11:**
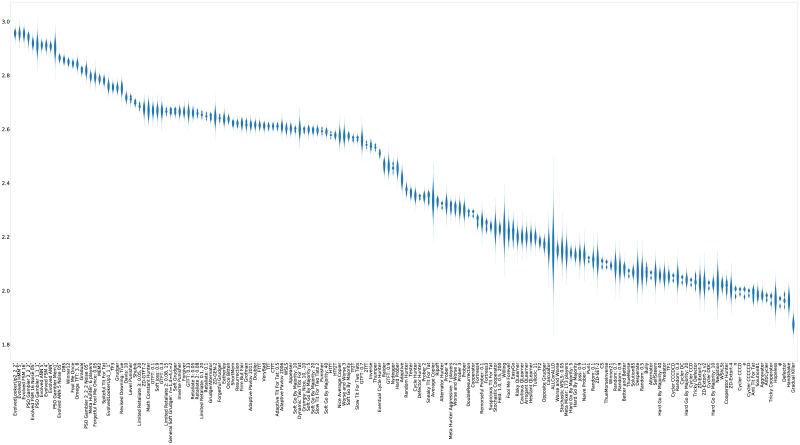
Standard tournament: Mean score per turn (strategies ordered by median score over 50000 tournaments).

For completeness, violin plots showing the distribution of the scores of each strategy (again ranked by median score) are shown in Fig 11.

**Fig 12:**
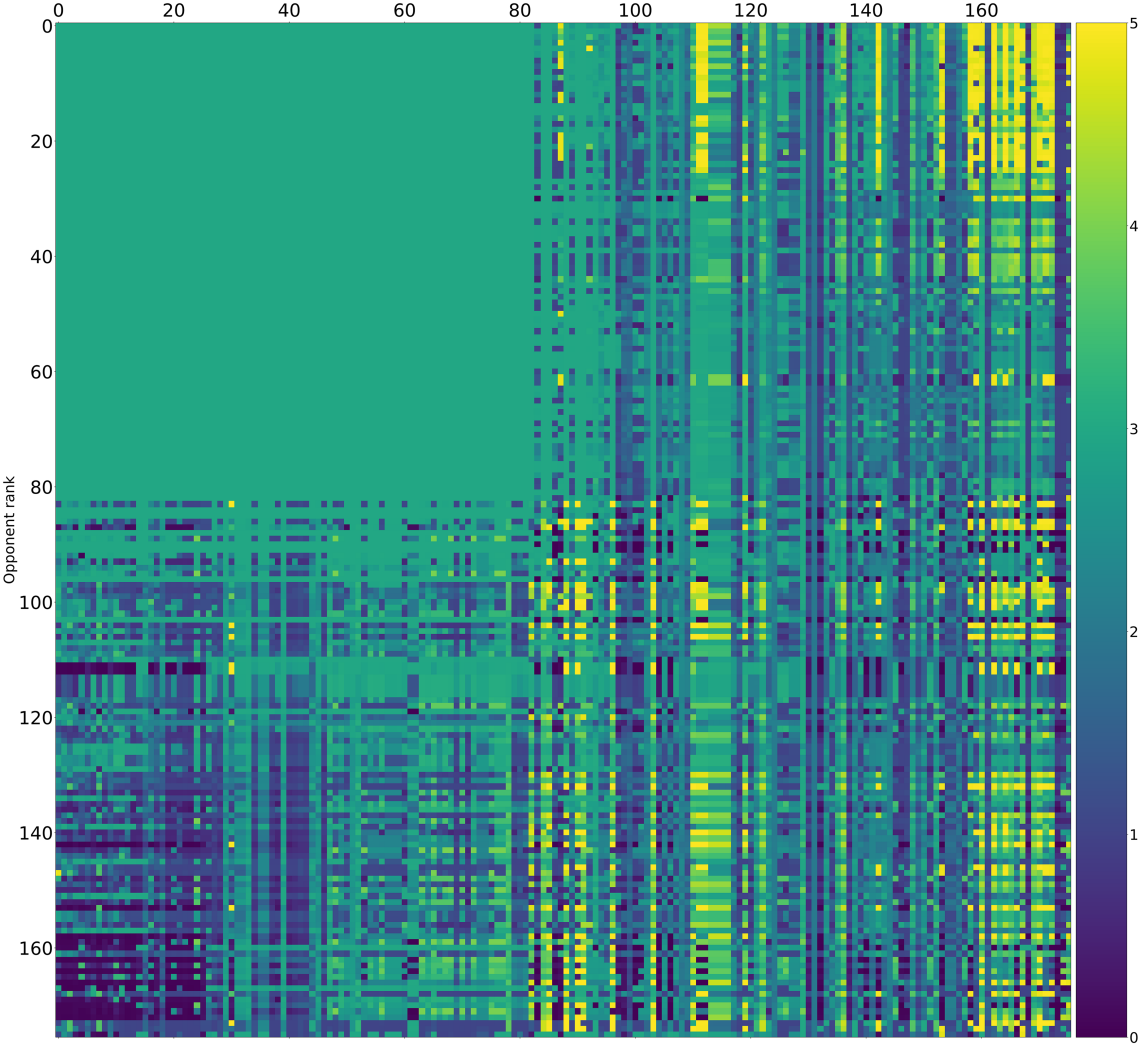
Standard tournament: Mean score per turn of row players against column players (ranked by median over 50000 tournaments).

Pairwise payoff results are given as a heatmap (Fig 12) which shows that many strategies achieve mutual cooperation (obtaining a score of 3). The top performing strategies never defect first yet are able to exploit weaker strategies that attempt to defect.

**Fig 13:**
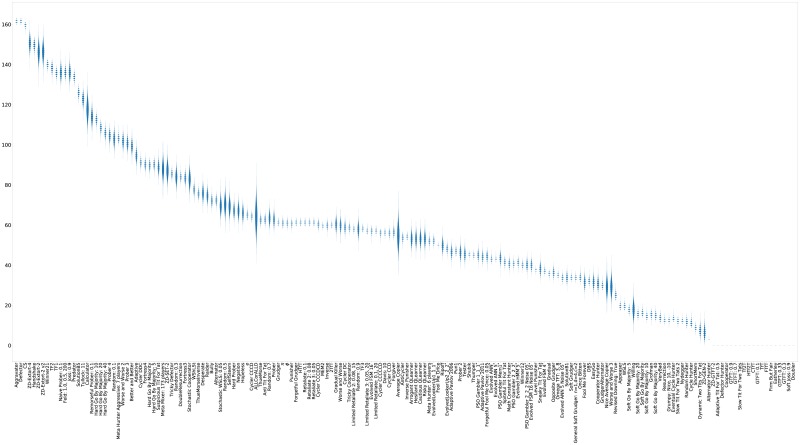
Standard tournament: Number of wins per tournament (ranked by median over 50000 tournaments).

The strategies that win the most matches (Table 3) are Defector [1] and Aggravater [3], followed by handshaking and zero determinant strategies [33]. This includes two handshaking strategies that were the result of training to maximize Moran process fixation (TF1 and TF2). No strategies were trained specifically to win matches. None of the top scoring strategies appear in the top 15 list of strategies ranked by match wins. This can be seen in Fig 13 where the distribution of the number of wins of each strategy is shown.

**Table 3:** Standard tournament: Number of wins per tournament of top 15 strategies (ranked by median wins over 50000 tournaments).

	mean	std	min	5%	25%	50%	75%	95%	max
Aggravater	161.595	0.862	160	160.0	161.0	162.0	162.0	163.0	163
Defector	161.605	0.864	160	160.0	161.0	162.0	162.0	163.0	163
CS	159.646	1.005	155	158.0	159.0	160.0	160.0	161.0	161
ZD-Extort-4	150.598	2.662	138	146.0	149.0	151.0	152.0	155.0	162
Handshake	149.552	1.754	142	147.0	148.0	150.0	151.0	152.0	154
ZD-Extort-2	146.094	3.445	129	140.0	144.0	146.0	148.0	152.0	160
ZD-Extort-2 v2	146.291	3.425	131	141.0	144.0	146.0	149.0	152.0	160
Winner21	139.946	1.225	136	138.0	139.0	140.0	141.0	142.0	143
TF2	138.240	1.700	130	135.0	137.0	138.0	139.0	141.0	143
TF1	135.692	1.408	130	133.0	135.0	136.0	137.0	138.0	140
Naive Prober: 0.1	136.016	2.504	127	132.0	134.0	136.0	138.0	140.0	147
Feld: 1.0, 0.5, 200	136.087	1.696	130	133.0	135.0	136.0	137.0	139.0	144
Joss: 0.9	136.015	2.503	126	132.0	134.0	136.0	138.0	140.0	146
Predator	133.718	1.385	129	131.0	133.0	134.0	135.0	136.0	138
SolutionB5	125.843	1.509	120	123.0	125.0	126.0	127.0	128.0	131

The number of wins of the top strategies of Table 2 are shown in Table 4. It is evident that although these strategies score highly they do not win many matches: the strategy with the most number of wins is the Evolved FSM 16 strategy that at most won 60 (60/175 ≈ 34%) matches in a given tournament.

**Table 4:** Standard tournament: Number of wins per tournament of top 15 strategies (ranked by median score over 50000 tournaments) * indicates that the strategy was trained.

	mean	std	min	5%	25%	50%	75%	95%	max
EvolvedLookerUp2_2_2*	48.259	1.336	43	46.0	47.0	48.0	49.0	50.0	53
Evolved HMM 5*	41.358	1.221	36	39.0	41.0	41.0	42.0	43.0	45
Evolved FSM 16*	56.978	1.099	51	55.0	56.0	57.0	58.0	59.0	60
PSO Gambler 2_2_2*	40.692	1.089	36	39.0	40.0	41.0	41.0	42.0	45
Evolved FSM 16 Noise 05*	40.070	1.673	34	37.0	39.0	40.0	41.0	43.0	47
PSO Gambler 1_1_1*	45.005	1.595	38	42.0	44.0	45.0	46.0	48.0	51
Evolved ANN 5*	43.224	0.674	41	42.0	43.0	43.0	44.0	44.0	47
Evolved FSM 4*	37.227	0.951	34	36.0	37.0	37.0	38.0	39.0	41
Evolved ANN*	43.100	1.021	40	42.0	42.0	43.0	44.0	45.0	48
PSO Gambler Mem1*	43.444	1.837	34	40.0	42.0	43.0	45.0	46.0	51
Evolved ANN 5 Noise 05*	33.711	1.125	30	32.0	33.0	34.0	34.0	35.0	38
DBS	32.329	1.198	28	30.0	32.0	32.0	33.0	34.0	38
Winner12	40.179	1.037	36	39.0	39.0	40.0	41.0	42.0	44
Fool Me Once	50.121	0.422	48	50.0	50.0	50.0	50.0	51.0	52
Omega TFT: 3, 8	35.157	0.859	32	34.0	35.0	35.0	36.0	37.0	39

**Fig 14:**
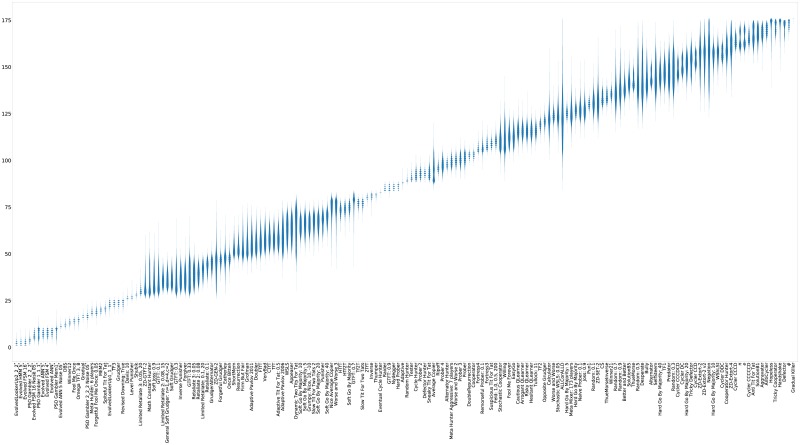
Standard tournament: Rank in each tournament (ranked by median over 50000 tournaments).

Finally, Table 5 and Fig 14 show the ranks (based on median score) of each strategy over the repeated tournaments. Whilst there is some stochasticity, the top three strategies almost always rank in the top three. For example, the worst that the EvolvedLookerUp_2_2_2 ranks in any tournament is 8th.

**Table 5:** Standard tournament: Rank in each tournament of top 15 strategies (ranked by median over 50000 tournaments) * indicates that the strategy was trained.

	mean	std	min	5%	25%	50%	75%	95%	max
EvolvedLookerUp2_2_2*	2.173	1.070	1	1.0	1.0	2.0	3.0	4.0	8
Evolved HMM 5*	2.321	1.275	1	1.0	1.0	2.0	3.0	5.0	10
Evolved FSM 16*	2.489	1.299	1	1.0	1.0	2.0	3.0	5.0	10
PSO Gambler 2_2_2*	3.961	1.525	1	2.0	3.0	4.0	5.0	7.0	10
Evolved FSM 16 Noise 05*	6.300	1.688	1	4.0	5.0	6.0	7.0	9.0	11
PSO Gambler 1_1_1*	7.082	2.499	1	3.0	5.0	7.0	9.0	10.0	17
Evolved ANN 5*	7.287	1.523	2	5.0	6.0	7.0	8.0	10.0	11
Evolved FSM 4*	7.527	1.631	2	5.0	6.0	8.0	9.0	10.0	12
Evolved ANN*	7.901	1.450	2	5.0	7.0	8.0	9.0	10.0	12
PSO Gambler Mem1*	8.222	2.535	1	4.0	6.0	9.0	10.0	12.0	20
Evolved ANN 5 Noise 05*	11.362	0.872	8	10.0	11.0	11.0	12.0	13.0	16
DBS	12.197	1.125	9	11.0	11.0	12.0	13.0	14.0	16
Winner12	13.221	1.137	9	11.0	12.0	13.0	14.0	15.0	17
Fool Me Once	13.960	1.083	9	12.0	13.0	14.0	15.0	15.0	17
Omega TFT: 3, 8	14.275	1.301	9	12.0	13.0	15.0	15.0	16.0	19

**Fig 15:**
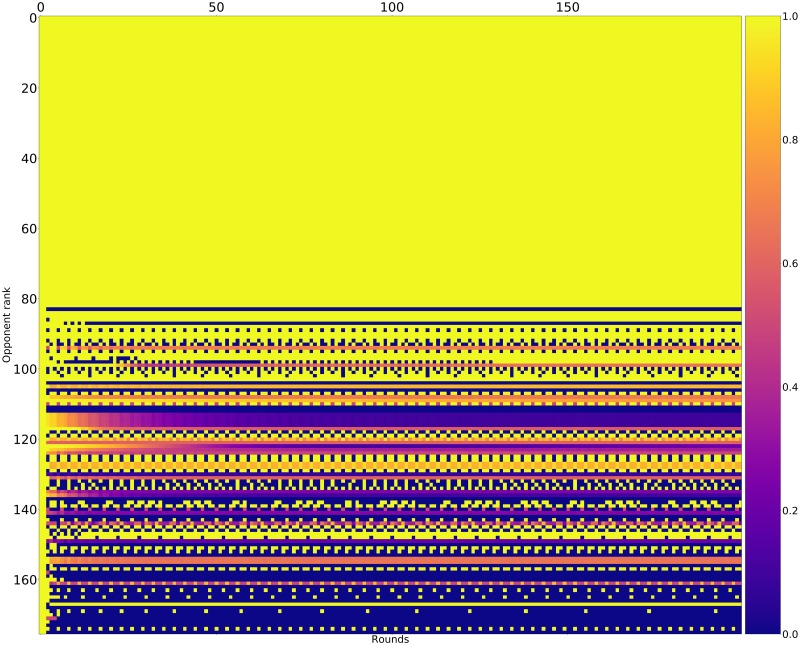
Cooperation rates for EvolvedLookerUp_2_2_2 (strategies ordered by median score over 10000 tournaments).

**Fig 16:**
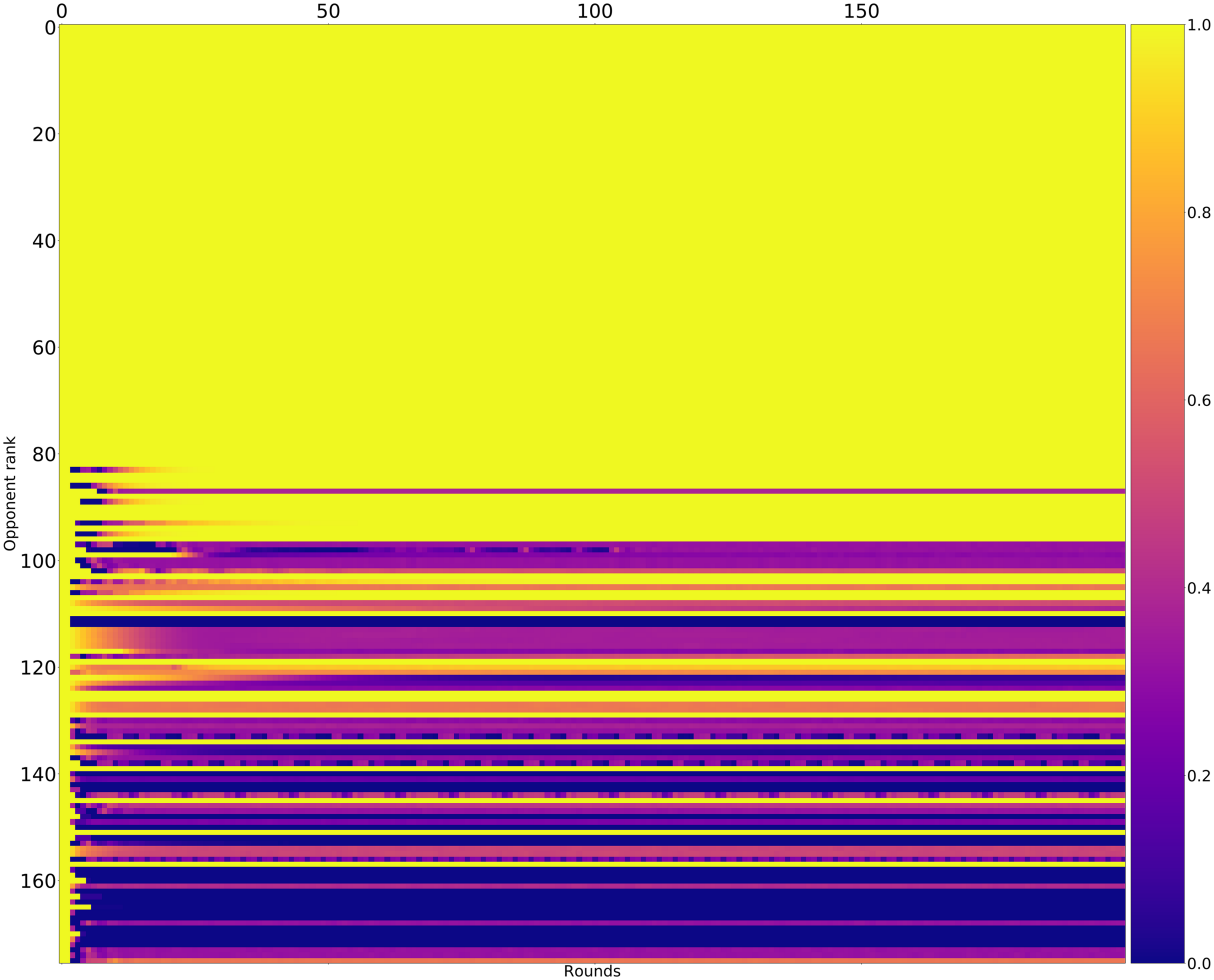
Cooperation rates for Evolved_HMM_5 (strategies ordered by median score over 10000 tournaments).

**Fig 17:**
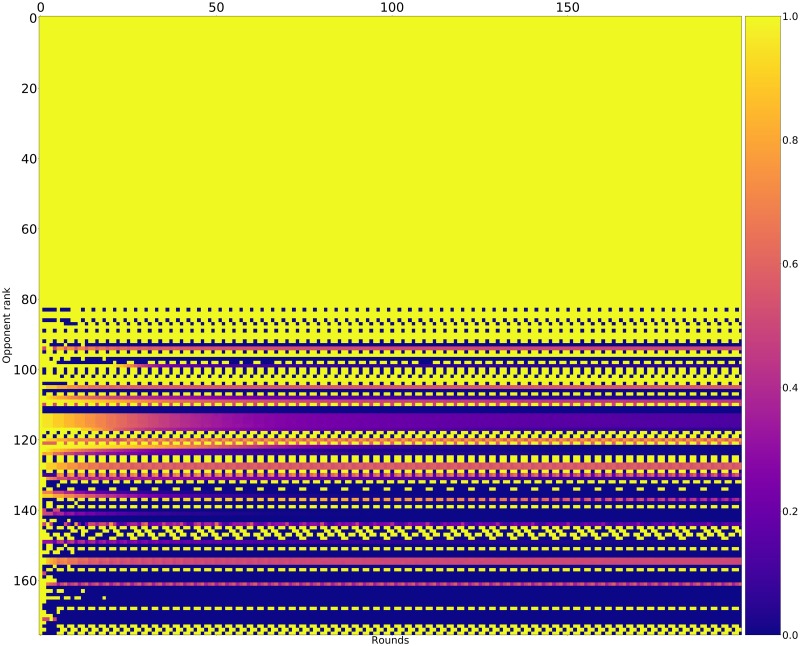
Cooperation rates for Evolved_FSM_16 (strategies ordered by median score over 10000 tournaments).

Figs 15–17 shows the rate of cooperation in each round for the top three strategies. The opponents in these figures are ordered according to performance by median score. It is evident that the high performing strategies share a common thread against the top strategies: they do not defect first and achieve mutual cooperation. Against the lower strategies they also do not defect first (a mean cooperation rate of 1 in the first round) but do learn to quickly retaliate.

### Noisy tournament

**Fig 18:**
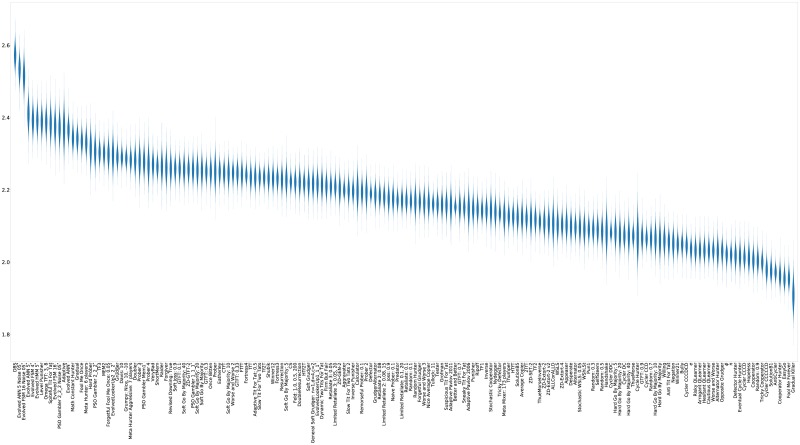
Noisy (5%) tournament: Mean score per turn (strategies ordered by median score over 50000 tournaments).

Results from noisy tournaments in which there is a 5% chance that an action is flipped are now described. As shown in Table 6 and Fig 18, the best performing strategies in median payoff are DBS, designed to account for noise, followed by two strategies trained in the presence of noise and three trained strategies trained without noise. One of the strategies trained with noise (PSO Gambler) actually performs less well than some of the other high ranking strategies including Spiteful TFT (TFT but defects indefinitely if the opponent defects twice consecutively) and OmegaTFT (also designed to handle noise). While DBS is the clear winner, it comes at a 6x increased run time over Evolved FSM 16 Noise 05.

**Table 6:** Noisy (5%) tournament: Mean score per turn of top 15 strategies (ranked by median over 50000 tournaments) * indicates that the strategy was trained.

	mean	std	min	5%	25%	50%	75%	95%	max
DBS	2.573	0.025	2.474	2.533	2.556	2.573	2.589	2.614	2.675
Evolved ANN 5 Noise 05*	2.534	0.025	2.418	2.492	2.517	2.534	2.551	2.575	2.629
Evolved FSM 16 Noise 05*	2.515	0.031	2.374	2.464	2.494	2.515	2.536	2.565	2.642
Evolved ANN 5*	2.410	0.030	2.273	2.359	2.389	2.410	2.430	2.459	2.536
Evolved FSM 4*	2.393	0.027	2.286	2.348	2.374	2.393	2.411	2.437	2.505
Evolved HMM 5*	2.392	0.026	2.289	2.348	2.374	2.392	2.409	2.435	2.493
Level Punisher	2.388	0.025	2.281	2.347	2.372	2.389	2.405	2.429	2.503
Omega TFT: 3, 8	2.387	0.026	2.270	2.344	2.370	2.388	2.405	2.430	2.498
Spiteful Tit For Tat	2.383	0.030	2.259	2.334	2.363	2.383	2.403	2.432	2.517
Evolved FSM 16*	2.375	0.029	2.239	2.326	2.355	2.375	2.395	2.423	2.507
PSO Gambler 2_2_2 Noise 05*	2.371	0.029	2.250	2.323	2.352	2.371	2.390	2.418	2.480
Adaptive	2.369	0.038	2.217	2.306	2.344	2.369	2.395	2.431	2.524
Evolved ANN*	2.365	0.022	2.270	2.329	2.351	2.366	2.380	2.401	2.483
Math Constant Hunter	2.344	0.022	2.257	2.308	2.329	2.344	2.359	2.382	2.445
Gradual	2.341	0.021	2.248	2.306	2.327	2.341	2.355	2.376	2.429

**Fig 19:**
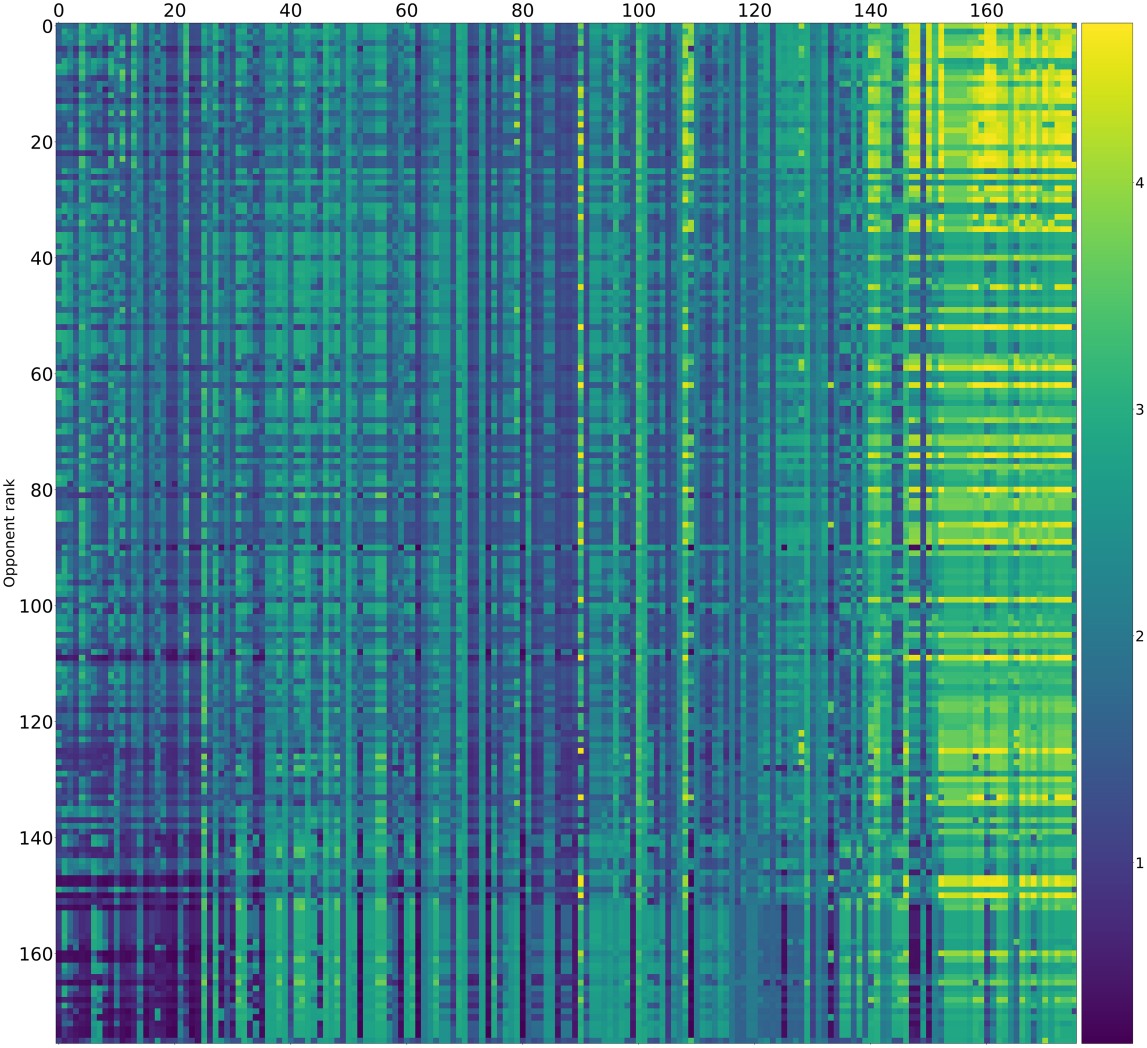
Noisy (5%) tournament: Mean score per turn of row players against column players (ranked by median over 50000 tournaments).

Recalling Table 2, the strategies trained in the presence of noise are also among the best performers in the absence of noise. As shown in Fig 19 the cluster of mutually cooperative strategies is broken by the noise at 5%. A similar collection of players excels at winning matches but again they have a poor total payoff.

**Fig 20:**
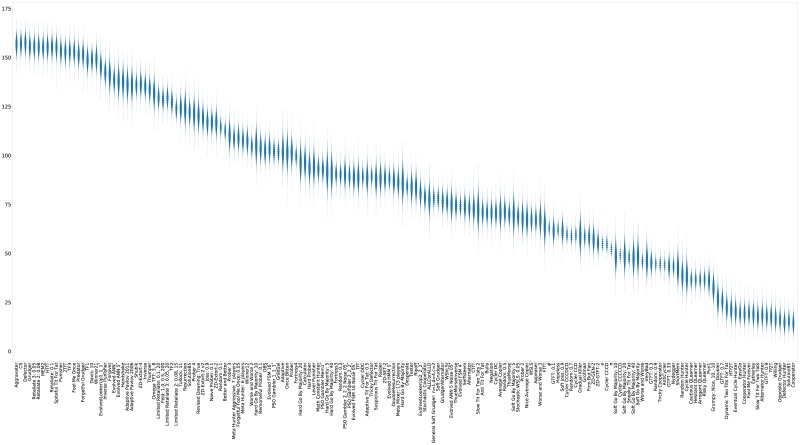
Noisy (5%) tournament: Number of wins per tournament (strategies ordered by median score over 50000 tournaments).

As shown in Table 7 and Fig 20 the strategies tallying the most wins are somewhat similar to the standard tournaments, with Defector, the handshaking CollectiveStrategy [34], and Aggravater appearing as the top three again.

**Table 7:** Noisy (5%) tournament: Number of wins per tournament of top 15 strategies (ranked by median wins over 50000 tournaments).

	mean	std	min	5%	25%	50%	75%	95%	max
Aggravater	156.654	3.328	141	151.0	154.0	157.0	159.0	162.0	170
CS	156.875	3.265	144	151.0	155.0	157.0	159.0	162.0	169
Defector	157.324	3.262	144	152.0	155.0	157.0	160.0	163.0	170
Grudger	155.590	3.303	143	150.0	153.0	156.0	158.0	161.0	168
Retaliate 3: 0.05	155.382	3.306	141	150.0	153.0	155.0	158.0	161.0	169
Retaliate 2: 0.08	155.365	3.320	140	150.0	153.0	155.0	158.0	161.0	169
MEM2	155.052	3.349	140	149.0	153.0	155.0	157.0	160.0	169
HTfT	155.298	3.344	141	150.0	153.0	155.0	158.0	161.0	168
Retaliate: 0.1	155.370	3.314	139	150.0	153.0	155.0	158.0	161.0	168
Spiteful Tit For Tat	155.030	3.326	133	150.0	153.0	155.0	157.0	160.0	167
Punisher	153.281	3.375	140	148.0	151.0	153.0	156.0	159.0	167
2TfT	152.823	3.429	138	147.0	151.0	153.0	155.0	158.0	165
TF3	153.031	3.327	138	148.0	151.0	153.0	155.0	158.0	166
Fool Me Once	152.817	3.344	138	147.0	151.0	153.0	155.0	158.0	166
Predator	151.406	3.403	138	146.0	149.0	151.0	154.0	157.0	165

As shown in Table 8, the top ranking strategies win a larger number of matches in the presence of noise. For example Spiteful Tit For Tat [35] in one tournament won almost all its matches (167).

**Table 8:** Noisy (5%) tournament: Number of wins per tournament of top 15 strategies (ranked by median score over 50000 tournaments) * indicates that the strategy was trained.

	mean	std	min	5%	25%	50%	75%	95%	max
DBS	102.545	3.671	87	97.0	100.0	103.0	105.0	109.0	118
Evolved ANN 5 Noise 05*	75.026	4.226	57	68.0	72.0	75.0	78.0	82.0	93
Evolved FSM 16 Noise 05*	88.699	3.864	74	82.0	86.0	89.0	91.0	95.0	104
Evolved ANN 5*	137.878	4.350	118	131.0	135.0	138.0	141.0	145.0	156
Evolved FSM 4*	74.250	2.694	64	70.0	72.0	74.0	76.0	79.0	85
Evolved HMM 5*	88.189	2.774	77	84.0	86.0	88.0	90.0	93.0	99
Level Punisher	94.263	4.789	75	86.0	91.0	94.0	97.0	102.0	116
Omega TFT: 3, 8	131.655	4.302	112	125.0	129.0	132.0	135.0	139.0	150
Spiteful Tit For Tat	155.030	3.326	133	150.0	153.0	155.0	157.0	160.0	167
Evolved FSM 16*	103.288	3.631	89	97.0	101.0	103.0	106.0	109.0	118
PSO Gambler 2_2_2 Noise 05*	90.515	4.012	75	84.0	88.0	90.0	93.0	97.0	109
Adaptive	101.898	4.899	83	94.0	99.0	102.0	105.0	110.0	124
Evolved ANN*	138.514	3.401	125	133.0	136.0	139.0	141.0	144.0	153
Math Constant Hunter	93.010	3.254	79	88.0	91.0	93.0	95.0	98.0	107
Gradual	101.899	2.870	91	97.0	100.0	102.0	104.0	107.0	114

**Fig 21:**
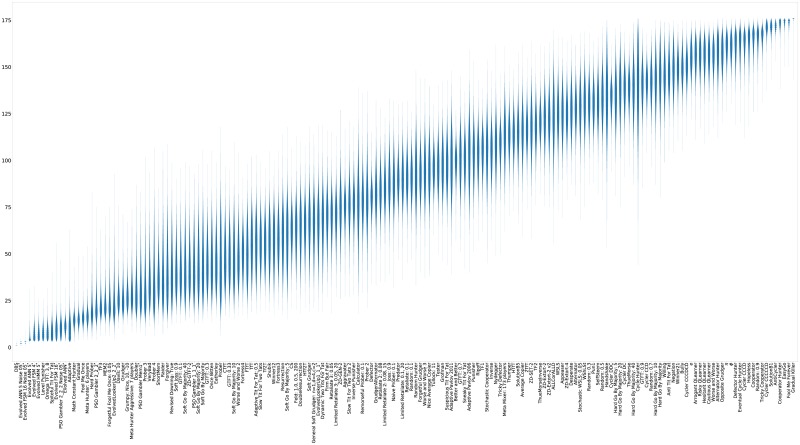
Noisy (5%) tournament: Rank in each tournament (strategies ordered by median score over 50000 tournaments).

Finally, Table 9 and Fig 21 show the ranks (based on median score) of each strategy over the repeated tournaments. We see that the stochasticity of the ranks understandably increases relative to the standard tournament. An exception is the top three strategies, for example, the DBS strategy never ranks lower than second and wins 75% of the time. The two strategies trained for noisy tournaments rank in the top three 95% of the time.

**Table 9:** Noisy (5%) tournament: Rank in each tournament of top 15 strategies (ranked by median over 50000 tournaments) * indicates that the strategy was trained.

	mean	std	min	5%	25%	50%	75%	95%	max
DBS	1.205	0.468	1	1.000	1.0	1.0	1.0	2.0	3
Evolved ANN 5 Noise 05*	2.184	0.629	1	1.000	2.0	2.0	3.0	3.0	5
Evolved FSM 16 Noise 05*	2.626	0.618	1	1.000	2.0	3.0	3.0	3.0	9
Evolved ANN 5*	6.371	2.786	2	4.000	4.0	5.0	8.0	12.0	31
Evolved FSM 4*	7.919	3.175	3	4.000	5.0	7.0	10.0	14.0	33
Evolved HMM 5*	7.996	3.110	3	4.000	6.0	7.0	10.0	14.0	26
Level Punisher	8.337	3.083	3	4.000	6.0	8.0	10.0	14.0	26
Omega TFT: 3, 8	8.510	3.249	3	4.000	6.0	8.0	11.0	14.0	32
Spiteful Tit For Tat	9.159	3.772	3	4.000	6.0	9.0	12.0	16.0	40
Evolved FSM 16*	10.218	4.099	3	4.975	7.0	10.0	13.0	17.0	56
PSO Gambler 2_2_2 Noise 05*	10.760	4.102	3	5.000	8.0	10.0	13.0	18.0	47
Evolved ANN*	11.346	3.252	3	6.000	9.0	11.0	13.0	17.0	32
Adaptive	11.420	5.739	3	4.000	7.0	11.0	14.0	21.0	63
Math Constant Hunter	14.668	3.788	3	9.000	12.0	15.0	17.0	21.0	43
Gradual	15.163	3.672	4	10.000	13.0	15.0	17.0	21.0	49

**Fig 22:**
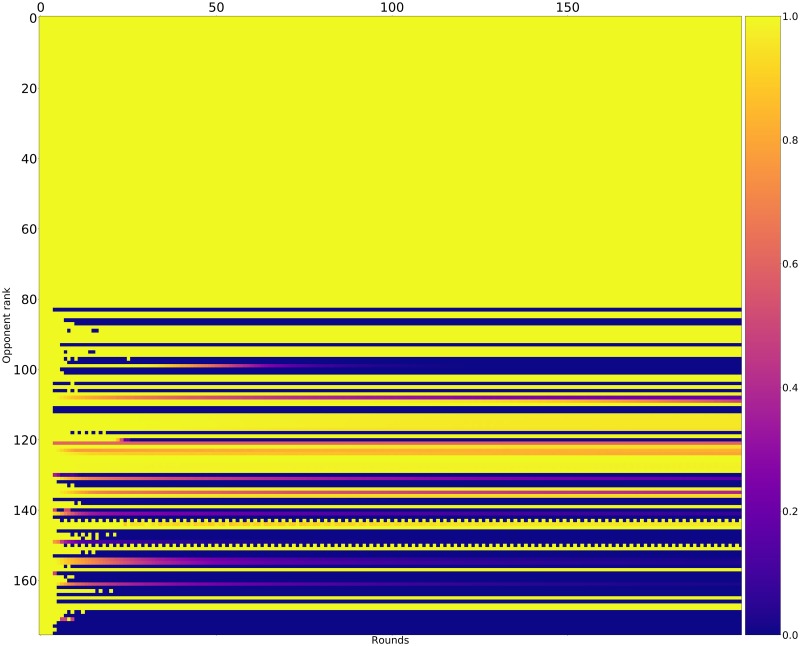
Cooperation rates for DBS (strategies ordered by median score over 10000 tournaments).

**Fig 23:**
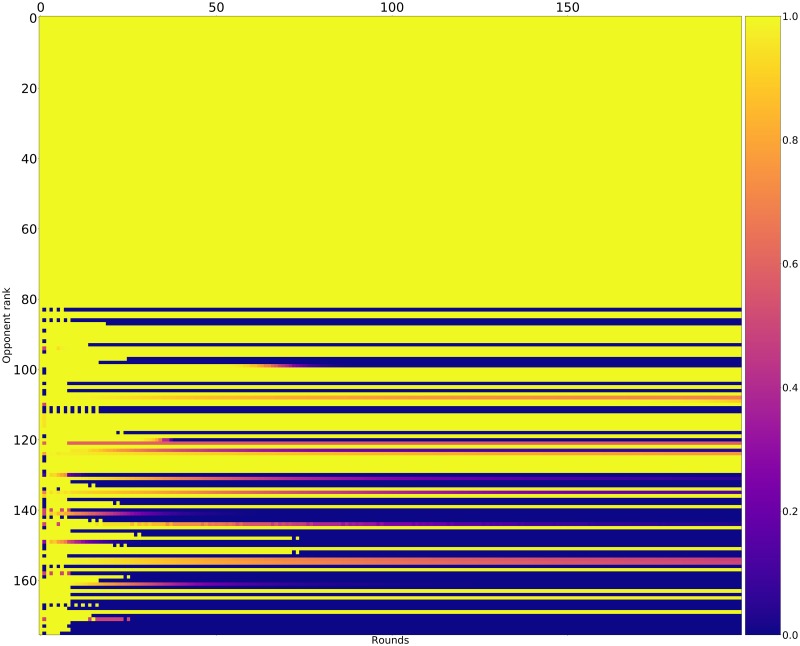
Cooperation rates for Evolved_ANN_5_Noise_05 (strategies ordered by median score over 10000 tournaments).

**Fig 24:**
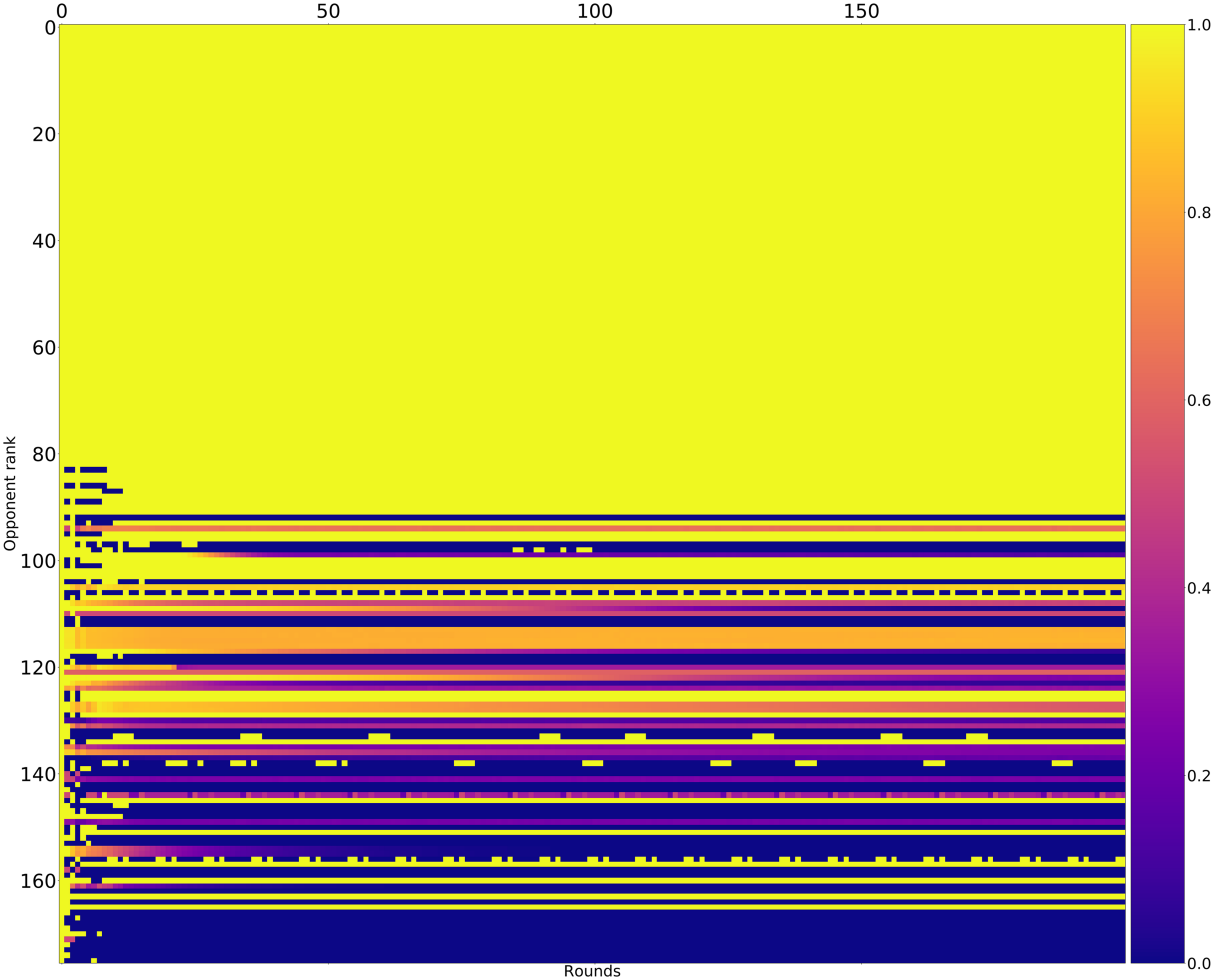
Cooperation rates for Evolved_FSM_16_Noise_05 (strategies ordered by median score over 10000 tournaments).

Figs 22–24 shows the rate of cooperation in each round for the top three strategies (in the absence of noise) and just as for the top performing strategies in the standard tournament it is evident that the strategies never defect first and learn to quickly punish poorer strategies.

‘

## Discussion

The tournament results indicate that pre-trained strategies are generally better than human designed strategies at maximizing payoff against a diverse set of opponents. An evolutionary algorithm produces strategies based on multiple generic archetypes that are able to achieve a higher average score than any other known opponent in a standard tournament. Most of the trained strategies use multiple rounds of the history of play (some using all of it) and outperform memory-one strategies from the literature. Interestingly, a trained memory one strategy produced by a particle swarm algorithm performs well, better than human designed strategies such as Win Stay Lose Shift and zero determinant strategies (which enforce a payoff difference rather than maximize total payoff).

In opposition to historical tournament results and community folklore, our results show that complex strategies can be effective for the IPD. Of all the human-designed strategies in the library, only DBS consistently performs well, and it is substantially more complex than traditional tournament winners like TFT, OmegaTFT, and zero determinant strategies.

The generic structure of the trained strategies did not appear to be critical for the standard tournament—strategies based on lookup tables, finite state machines, neural networks, and stochastic variants all performed well. Single layer neural networks performed well in both noisy and standard tournaments though these had some aspect of human involvement in the selection of features. This is in line with the other strategies also where some human decisions are made regarding the structure. For the LookerUp and Gambler archetypes a decision has to be made regarding the number of rounds of history and initial play that are to be used. In contrast, the finite state machines and hidden Markov models required only a choice of the number of states, and the training algorithm can eliminate unneeded states in the case of finite state machines (evidenced by the unconnected nodes in the diagrams for the included representations).

Many strategies can be represented by multiple archetypes, however some archetypes will be more efficient in encoding the patterns present in the data. The fact that the Lookerup strategy does the best for the standard tournament indicates that it represents an efficient reduction of dimension which in turn makes its training more efficient. In particular the first rounds of play were valuable bits of information. For the noisy tournament however the dimension reduction represented by some archetypes indicates that some features of the data are not captured by the lookup tables while they are by the neural networks and the finite state machines, allowing the latter to adapt better to the noisy environment. Intuitively, a noisy environment can significantly affect a lookup table based on the last two rounds of play since these action pairs compete with probing defections, apologies, and retaliations. Accordingly, it is not surprising that additional parameter space is needed to adapt to a noisy environment.

Two strategies designed specifically to account for noise, DBS and OmegaTFT, perform well and only DBS performs better than the trained strategies and **only** in noisy contexts. Empirically we find that DBS (with its default parameters) does not win tournaments at 1% noise. However DBS has a parameter that accounts for the expected amount of noise and a followup study with various noise levels could make a more complete study of the performance of DBS and strategies trained at various noise levels.

The strategies trained to maximize their average score are generally cooperative and do not defect first. Maximizing for individual performance across a collection of opponents leads to mutual cooperation despite the fact that mutual cooperation is an unstable evolutionary equilibrium for the prisoner’s dilemma. Specifically it is noted that the reinforcement learning process for maximizing payoff does not lead to exploitative zero determinant strategies, which may also be a result of the collection of training strategies, of which several retaliate harshly. Training with the objective of maximizing payoff difference may produce strategies more like zero determinant strategies.

For the trained strategies utilizing look up tables we generally found those that incorporate one or more of the initial rounds of play outperformed those that did not. The strategies based on neural networks and finite state machines also are able to condition throughout a match on the first rounds of play. Accordingly, we conclude that first impressions matter in the IPD. The best strategies are nice (never defecting first) and the impact of the first rounds of play could be further investigated with the Axelrod library in future work by e.g. forcing all strategies to defect on the first round.

We note that as the library grows, the top performing strategies sometimes shuffle, and are not retrained automatically. Most of the strategies were trained on an earlier version of the library (v2.2.0: [36]) that did not include DBS and several other opponents. The precise parameters that are optimal will depend on the pool of opponents. Moreover we have not extensively trained strategies to determine the minimum parameter spaces that are sufficient—neural networks with fewer nodes and features and finite state machines with fewer states may suffice. See [37] for discussion of resource availability for IPD strategies.

Finally, whilst we have considered the robustness of our claims and results with respect to noise it would also be of interest to train strategies for different versions of the stage game (also referred to as *dilemma strength*) [38, 39]. Our findings seems to indicate that obtaining strong strategies for other games through reinforcement learning would be possible.

## A Appendix A: List of players

The players used for this study are from Axelrod version 2.13.0 [3].

1. *ϕ*—*Deterministic*—*Memory depth*: ∞. [3]
2. *π*—*Deterministic*—*Memory depth*: ∞. [3]
3. *e*—*Deterministic*—*Memory depth*: ∞. [3]
4. ALLCorALLD—*Stochastic*—*Memory depth*: 1. [3]
5. Adaptive—*Deterministic*—*Memory depth*: ∞. [43]
6. Adaptive Pavlov 2006—*Deterministic*—*Memory depth*: ∞. [12]
7. Adaptive Pavlov 2011—*Deterministic*—*Memory depth*: ∞. [43]
8. Adaptive Tit For Tat: 0.5—*Deterministic*—*Memory depth*: ∞. [44]
9. Aggravater—*Deterministic*—*Memory depth*: ∞. [3]
10. Alternator—*Deterministic*—*Memory depth*: 1. [1, 45]
11. Alternator Hunter—*Deterministic*—*Memory depth*: ∞. [3]
12. Anti Tit For Tat—*Deterministic*—*Memory depth*: 1. [46]
13. AntiCycler—*Deterministic*—*Memory depth*: ∞. [3]
14. Appeaser—*Deterministic*—*Memory depth*: ∞. [3]
15. Arrogant QLearner—*Stochastic*—*Memory depth*: ∞. [3]
16. Average Copier—*Stochastic*—*Memory depth*: ∞. [3]
17. Better and Better—*Stochastic*—*Memory depth*: ∞. [35]
18. Bully—*Deterministic*—*Memory depth*: 1. [47]
19. Calculator—*Stochastic*—*Memory depth*: ∞. [35]
20. Cautious QLearner—*Stochastic*—*Memory depth*: ∞. [3]
21. CollectiveStrategy (**CS**)—*Deterministic*—*Memory depth*: ∞. [34]
22. Contrite Tit For Tat (**CTfT**)—*Deterministic*—*Memory depth*: 3. [48]
23. Cooperator—*Deterministic*—*Memory depth*: 0. [1, 33, 45]
24. Cooperator Hunter—*Deterministic*—*Memory depth*: ∞. [3]
25. Cycle Hunter—*Deterministic*—*Memory depth*: ∞. [3]
26. Cycler CCCCCD—*Deterministic*—*Memory depth*: 5. [3]
27. Cycler CCCD—*Deterministic*—*Memory depth*: 3. [3]
28. Cycler CCCDCD—*Deterministic*—*Memory depth*: 5. [3]
29. Cycler CCD—*Deterministic*—*Memory depth*: 2. [45]
30. Cycler DC—*Deterministic*—*Memory depth*: 1. [3]
31. Cycler DDC—*Deterministic*—*Memory depth*: 2. [45]
32. DBS: 0.75, 3, 4, 3, 5—*Deterministic*—*Memory depth*: ∞. [32]
33. Davis: 10—*Deterministic*—*Memory depth*: ∞. [25]
34. Defector—*Deterministic*—*Memory depth*: 0. [1, 33, 45]
35. Defector Hunter—*Deterministic*—*Memory depth*: ∞. [3]
36. Desperate—*Stochastic*—*Memory depth*: 1. [49]
37. DoubleResurrection—*Deterministic*—*Memory depth*: 5. [50]
38. Doubler—*Deterministic*—*Memory depth*: ∞. [35]
39. Dynamic Two Tits For Tat—*Stochastic*—*Memory depth*: 2. [3]
40. EasyGo—*Deterministic*—*Memory depth*: ∞. [35, 43]
41. Eatherley—*Stochastic*—*Memory depth*: ∞. [10]
42. Eventual Cycle Hunter—*Deterministic*—*Memory depth*: ∞. [3]
43. Evolved ANN—*Deterministic*—*Memory depth*: ∞. [3]
44. Evolved ANN 5—*Deterministic*—*Memory depth*: ∞. [3]
45. Evolved ANN 5 Noise 05—*Deterministic*—*Memory depth*: ∞. [3]
46. Evolved FSM 16—*Deterministic*—*Memory depth*: 16. [3]
47. Evolved FSM 16 Noise 05—*Deterministic*—*Memory depth*: 16. [3]
48. Evolved FSM 4—*Deterministic*—*Memory depth*: 4. [3]
49. Evolved HMM 5—*Stochastic*—*Memory depth*: 5. [3]
50. EvolvedLookerUp1_1_1—*Deterministic*—*Memory depth*: ∞. [3]
51. EvolvedLookerUp2_2_2—*Deterministic*—*Memory depth*: ∞. [3]
52. Feld: 1.0, 0.5, 200—*Stochastic*—*Memory depth*: 200. [25]
53. Firm But Fair—*Stochastic*—*Memory depth*: 1. [51]
54. Fool Me Forever—*Deterministic*—*Memory depth*: ∞. [3]
55. Fool Me Once—*Deterministic*—*Memory depth*: ∞. [3]
56. Forgetful Fool Me Once: 0.05—*Stochastic*—*Memory depth*: ∞. [3]
57. Forgetful Grudger—*Deterministic*—*Memory depth*: 10. [3]
58. Forgiver—*Deterministic*—*Memory depth*: ∞. [3]
59. Forgiving Tit For Tat (**FTfT**)—*Deterministic*—*Memory depth*: ∞. [3]
60. Fortress3—*Deterministic*—*Memory depth*: 3. [14]
61. Fortress4—*Deterministic*—*Memory depth*: 4. [14]
62. GTFT: 0.1—*Stochastic*—*Memory depth*: 1.
63. GTFT: 0.3—*Stochastic*—*Memory depth*: 1.
64. GTFT: 0.33—*Stochastic*—*Memory depth*: 1. [23, 52]
65. GTFT: 0.7—*Stochastic*—*Memory depth*: 1.
66. GTFT: 0.9—*Stochastic*—*Memory depth*: 1.
67. General Soft Grudger: n = 1, d = 4, c = 2—*Deterministic*—*Memory depth*: ∞. [3]
68. Gradual—*Deterministic*—*Memory depth*: ∞. [53]
69. Gradual Killer: (‘D’, ‘D’, ‘D’, ‘D’, ‘D’, ‘C’, ‘C’)—*Deterministic*—*Memory depth*: ∞. [35]
70. Grofman—*Stochastic*—*Memory depth*: ∞. [25]
71. Grudger—*Deterministic*—*Memory depth*: 1. [25, 43, 49, 53, 54]
72. GrudgerAlternator—*Deterministic*—*Memory depth*: ∞. [35]
73. Grumpy: Nice, 10, −10—*Deterministic*—*Memory depth*: ∞. [3]
74. Handshake—*Deterministic*—*Memory depth*: ∞. [55]
75. Hard Go By Majority—*Deterministic*—*Memory depth*: ∞. [45]
76. Hard Go By Majority: 10—*Deterministic*—*Memory depth*: 10. [3]
77. Hard Go By Majority: 20—*Deterministic*—*Memory depth*: 20. [3]
78. Hard Go By Majority: 40—*Deterministic*—*Memory depth*: 40. [3]
79. Hard Go By Majority: 5—*Deterministic*—*Memory depth*: 5. [3]
80. Hard Prober—*Deterministic*—*Memory depth*: ∞. [35]
81. Hard Tit For 2 Tats (**HTf2T**)—*Deterministic*—*Memory depth*: 3. [7]
82. Hard Tit For Tat (**HTfT**)—*Deterministic*—*Memory depth*: 3. [56]
83. Hesitant QLearner—*Stochastic*—*Memory depth*: ∞. [3]
84. Hopeless—*Stochastic*—*Memory depth*: 1. [49]
85. Inverse—*Stochastic*—*Memory depth*: ∞. [3]
86. Inverse Punisher—*Deterministic*—*Memory depth*: ∞. [3]
87. Joss: 0.9—*Stochastic*—*Memory depth*: 1. [7, 25]
88. Level Punisher—*Deterministic*—*Memory depth*: ∞. [50]
89. Limited Retaliate 2: 0.08, 15—*Deterministic*—*Memory depth*: ∞. [3]
90. Limited Retaliate 3: 0.05, 20—*Deterministic*—*Memory depth*: ∞. [3]
91. Limited Retaliate: 0.1, 20—*Deterministic*—*Memory depth*: ∞. [3]
92. MEM2—*Deterministic*—*Memory depth*: ∞. [57]
93. Math Constant Hunter—*Deterministic*—*Memory depth*: ∞. [3]
94. Meta Hunter Aggressive: 7 players—*Deterministic*—*Memory depth*: ∞. [3]
95. Meta Hunter: 6 players—*Deterministic*—*Memory depth*: ∞. [3]
96. Meta Mixer: 173 players—*Stochastic*—*Memory depth*: ∞. [3]
97. Naive Prober: 0.1—*Stochastic*—*Memory depth*: 1. [43]
98. Negation—*Stochastic*—*Memory depth*: 1. [56]
99. Nice Average Copier—*Stochastic*—*Memory depth*: ∞. [3]
100. Nydegger—*Deterministic*—*Memory depth*: 3. [25]
101. Omega TFT: 3, 8—*Deterministic*—*Memory depth*: ∞. [12]
102. Once Bitten—*Deterministic*—*Memory depth*: 12. [3]
103. Opposite Grudger—*Deterministic*—*Memory depth*: ∞. [3]
104. PSO Gambler 1_1_1—*Stochastic*—*Memory depth*: ∞. [3]
105. PSO Gambler 2_2_2—*Stochastic*—*Memory depth*: ∞. [3]
106. PSO Gambler 2_2_2 Noise 05—*Stochastic*—*Memory depth*: ∞. [3]
107. PSO Gambler Mem1—*Stochastic*—*Memory depth*: 1. [3]
108. Predator—*Deterministic*—*Memory depth*: 9. [14]
109. Prober—*Deterministic*—*Memory depth*: ∞. [43]
110. Prober 2—*Deterministic*—*Memory depth*: ∞. [35]
111. Prober 3—*Deterministic*—*Memory depth*: ∞. [35]
112. Prober 4—*Deterministic*—*Memory depth*: ∞. [35]
113. Pun1—*Deterministic*—*Memory depth*: 2. [14]
114. Punisher—*Deterministic*—*Memory depth*: ∞. [3]
115. Raider—*Deterministic*—*Memory depth*: 3. [17]
116. Random Hunter—*Deterministic*—*Memory depth*: ∞. [3]
117. Random: 0.1—*Stochastic*—*Memory depth*: 0.
118. Random: 0.3—*Stochastic*—*Memory depth*: 0.
119. Random: 0.5—*Stochastic*—*Memory depth*: 0. [25, 44]
120. Random: 0.7—*Stochastic*—*Memory depth*: 0.
121. Random: 0.9—*Stochastic*—*Memory depth*: 0.
122. Remorseful Prober: 0.1—*Stochastic*—*Memory depth*: 2. [43]
123. Resurrection—*Deterministic*—*Memory depth*: 5. [50]
124. Retaliate 2: 0.08—*Deterministic*—*Memory depth*: ∞. [3]
125. Retaliate 3: 0.05—*Deterministic*—*Memory depth*: ∞. [3]
126. Retaliate: 0.1—*Deterministic*—*Memory depth*: ∞. [3]
127. Revised Downing: True—*Deterministic*—*Memory depth*: ∞. [25]
128. Ripoff—*Deterministic*—*Memory depth*: 2. [58]
129. Risky QLearner—*Stochastic*—*Memory depth*: ∞. [3]
130. SelfSteem—*Stochastic*—*Memory depth*: ∞. [59]
131. ShortMem—*Deterministic*—*Memory depth*: 10. [59]
132. Shubik—*Deterministic*—*Memory depth*: ∞. [25]
133. Slow Tit For Two Tats—*Deterministic*—*Memory depth*: 2. [3]
134. Slow Tit For Two Tats 2—*Deterministic*—*Memory depth*: 2. [35]
135. Sneaky Tit For Tat—*Deterministic*—*Memory depth*: ∞. [3]
136. Soft Go By Majority—*Deterministic*—*Memory depth*: ∞. [1, 45]
137. Soft Go By Majority: 10—*Deterministic*—*Memory depth*: 10. [3]
138. Soft Go By Majority: 20—*Deterministic*—*Memory depth*: 20. [3]
139. Soft Go By Majority: 40—*Deterministic*—*Memory depth*: 40. [3]
140. Soft Go By Majority: 5—*Deterministic*—*Memory depth*: 5. [3]
141. Soft Grudger—*Deterministic*—*Memory depth*: 6. [43]
142. Soft Joss: 0.9—*Stochastic*—*Memory depth*: 1. [35]
143. SolutionB1—*Deterministic*—*Memory depth*: 3. [15]
144. SolutionB5—*Deterministic*—*Memory depth*: 5. [15]
145. Spiteful Tit For Tat—*Deterministic*—*Memory depth*: ∞. [35]
146. Stochastic Cooperator—*Stochastic*—*Memory depth*: 1. [60]
147. Stochastic WSLS: 0.05—*Stochastic*—*Memory depth*: 1. [3]
148. Suspicious Tit For Tat—*Deterministic*—*Memory depth*: 1. [46, 53]
149. TF1—*Deterministic*—*Memory depth*: ∞. [3]
150. TF2—*Deterministic*—*Memory depth*: ∞. [3]
151. TF3—*Deterministic*—*Memory depth*: ∞. [3]
152. Tester—*Deterministic*—*Memory depth*: ∞. [10]
153. ThueMorse—*Deterministic*—*Memory depth*: ∞. [3]
154. ThueMorseInverse—*Deterministic*—*Memory depth*: ∞. [3]
155. Thumper—*Deterministic*—*Memory depth*: 2. [58]
156. Tit For 2 Tats (**Tf2T**)—*Deterministic*—*Memory depth*: 2. [1]
157. Tit For Tat (**TfT**)—*Deterministic*—*Memory depth*: 1. [25]
158. Tricky Cooperator—*Deterministic*—*Memory depth*: 10. [3]
159. Tricky Defector—*Deterministic*—*Memory depth*: ∞. [3]
160. Tullock: 11—*Stochastic*—*Memory depth*: 11. [25]
161. Two Tits For Tat (**2TfT**)—*Deterministic*—*Memory depth*: 2. [1]
162. VeryBad—*Deterministic*—*Memory depth*: ∞. [59]
163. Willing—*Stochastic*—*Memory depth*: 1. [49]
164. Win-Shift Lose-Stay: D (**WShLSt**)—*Deterministic*—*Memory depth*: 1. [43]
165. Win-Stay Lose-Shift: C (**WSLS**)—*Deterministic*—*Memory depth*: 1. [7, 52, 61]
166. Winner12—*Deterministic*—*Memory depth*: 2. [26]
167. Winner21—*Deterministic*—*Memory depth*: 2. [26]
168. Worse and Worse—*Stochastic*—*Memory depth*: ∞. [35]
169. Worse and Worse 2—*Stochastic*—*Memory depth*: ∞. [35]
170. Worse and Worse 3—*Stochastic*—*Memory depth*: ∞. [35]
171. ZD-Extort-2 v2: 0.125, 0.5, 1—*Stochastic*—*Memory depth*: 1. [62]
172. ZD-Extort-2: 0.1111111111111111, 0.5—*Stochastic*—*Memory depth*: 1. [7]
173. ZD-Extort-4: 0.23529411764705882, 0.25, 1—*Stochastic*—*Memory depth*: 1. [3]
174. ZD-GEN-2: 0.125, 0.5, 3—*Stochastic*—*Memory depth*: 1. [62]
175. ZD-GTFT-2: 0.25, 0.5—*Stochastic*—*Memory depth*: 1. [7]
176. ZD-SET-2: 0.25, 0.0, 2—*Stochastic*—*Memory depth*: 1. [62]

## References

[1] Axelrod RM. The evolution of cooperation. Basic books; 2006.

[2] Knight V, Campbell O, Harper M, Langner K, Campbell J, Campbell T, et al An Open Framework for the Reproducible Study of the Iterated Prisoner’s Dilemma. Journal of Open Research Software. 2016;4(1).

[3] project developers TA. Axelrod-Python/Axelrod: v2.13.0; 2017. https://doi.org/10.5281/zenodo.801749.

[4] Nowak M, Sigmund K. A strategy of win-stay, lose-shift that outperforms tit-for-tat in the Prisoner’s Dilemma game. Nature. 1993;364(6432):56 doi: 10.1038/364056a0 8316296 10.1038/364056a0

[5] Slany W, Kienreich W. On some winning strategies for the Iterated Prisoner’s Dilemma, or, Mr. Nice Guy and the Cosa Nostra. The Iterated Prisoners’ Dilemma: 20 Years on. 2007;4:171 doi: 10.1142/9789812770684_0008

[6] Li J. How to design a strategy to win an IPD tournament. The iterated prisoner’s dilemma. 2007;20:89–104. doi: 10.1142/9789812770684_0004

[7] Stewart AJ, Plotkin JB. Extortion and cooperation in the Prisoner’s Dilemma. Proceedings of the National Academy of Sciences. 2012;109(26):10134–10135. doi: 10.1073/pnas.1208087109 10.1073/pnas.1208087109 PMC3387035 22711812

[8] Axelrod R. Effective Choice in the Prisoner’s Dilemma. Journal of Conflict Resolution. 1980;24(1):3–25. doi: 10.1177/002200278002400101

[9] Bendor J, Kramer RM, Stout S. When in doubt…: Cooperation in a noisy prisoner’s dilemma. Journal of Conflict Resolution. 1991;35(4):691–719. doi: 10.1177/0022002791035004007

[10] Axelrod R. More Effective Choice in the Prisoner’s Dilemma. Journal of Conflict Resolution. 1980;24(3):379–403. doi: 10.1177/002200278002400301

[11] Stephens DW, McLinn CM, Stevens JR. Discounting and reciprocity in an Iterated Prisoner’s Dilemma. Science (New York, NY). 2002;298(5601):2216–2218. doi: 10.1126/science.1078498 10.1126/science.1078498 12481142

[12] Kendall G, Yao X, Chong SY. The iterated prisoners’ dilemma: 20 years on. vol. 4 World Scientific; 2007.

[13] Ashlock D. Training function stacks to play the iterated prisoner’s dilemma. In: Computational Intelligence and Games, 2006 IEEE Symposium on. IEEE; 2006. p. 111–118.

[14] Ashlock W, Ashlock D. Changes in prisoner’s dilemma strategies over evolutionary time with different population sizes. In: Evolutionary Computation, 2006. CEC 2006. IEEE Congress on. IEEE; 2006. p. 297–304.

[15] Ashlock D, Brown JA, Hingston P. Multiple Opponent Optimization of Prisoner’s Dilemma Playing Agents. IEEE Transactions on Computational Intelligence and AI in Games. 2015;7(1):53–65. doi: 10.1109/TCIAIG.2014.2326012

[16] Ashlock W, Ashlock D. Shaped prisoner’s dilemma automata. In: Computational Intelligence and Games (CIG), 2014 IEEE Conference on. IEEE; 2014. p. 1–8.

[17] Ashlock W, Tsang J, Ashlock D. The evolution of exploitation. In: Foundations of Computational Intelligence (FOCI), 2014 IEEE Symposium on. IEEE; 2014. p. 135–142.

[18] Barlow LA, Ashlock D. Varying decision inputs in Prisoner’s Dilemma. In: Computational Intelligence in Bioinformatics and Computational Biology (CIBCB), 2015 IEEE Conference on. IEEE; 2015. p. 1–8.

[19] Fogel DB. Evolving behaviors in the iterated prisoner’s dilemma. Evolutionary Computation. 1993;1(1):77–97. doi: 10.1162/evco.1993.1.1.77 10.1162/evco_a_00322 36976882

[20] Marks RE. Niche strategies: the Prisoner’s Dilemma computer tournaments revisited. In: JOURNAL OF EVOLUTIONARY ECONOMICS. Citeseer; 1989.

[21] Sudo T, Goto K, Nojima Y, Ishibuchi H. Effects of ensemble action selection with different usage of player’s memory resource on the evolution of cooperative strategies for iterated prisoner’s dilemma game. In: Evolutionary Computation (CEC), 2015 IEEE Congress on. IEEE; 2015. p. 1505–1512.

[22] Vassiliades V, Christodoulou C. Multiagent reinforcement learning in the iterated prisoner’s dilemma: fast cooperation through evolved payoffs. In: Neural Networks (IJCNN), The 2010 International Joint Conference on. IEEE; 2010. p. 1–8.

[23] Gaudesi M, Piccolo E, Squillero G, Tonda A. Exploiting evolutionary modeling to prevail in iterated prisoner’s dilemma tournaments. IEEE Transactions on Computational Intelligence and AI in Games. 2016;8(3):288–300. doi: 10.1109/TCIAIG.2015.2439061

[24] Franken N, Engelbrecht AP. Particle swarm optimization approaches to coevolve strategies for the iterated prisoner’s dilemma. IEEE Transactions on Evolutionary Computation. 2005;9(6):562–579. doi: 10.1109/TEVC.2005.856202

[25] Axelrod R. Effective choice in the prisoner’s dilemma. Journal of conflict resolution. 1980;24(1):3–25. doi: 10.1177/002200278002400101

[26] Mathieu P, Delahaye JP. New Winning Strategies for the Iterated Prisoner’s Dilemma (Extended Abstract). 14th International Conference on Autonomous Agents and Multiagent Systems (AAMAS 2015). 2015; p. 1665–1666.

[27] Turing AM. Computing machinery and intelligence. Mind. 1950;59(236):433–460. doi: 10.1093/mind/LIX.236.433

[28] Imran M, Hashim R, Khalid NEA. An overview of particle swarm optimization variants. Procedia Engineering. 2013;53:491–496. doi: 10.1016/j.proeng.2013.02.063

[29] Moriarty DE, Schultz AC, Grefenstette JJ. Evolutionary algorithms for reinforcement learning. J Artif Intell Res(JAIR). 1999;11:241–276.

[30] Harper M, Knight V, Jones M, Koutsovoulos G. Axelrod-Python/axelrod-dojo: V0.0.2; 2017. https://doi.org/10.5281/zenodo.832282.

[31] Knight V, Harper M. Data for: Reinforcement Learning Produces Dominant Strategies for the Iterated Prisoner’s Dilemma; 2017. https://doi.org/10.5281/zenodo.832287. 10.1371/journal.pone.0188046 PMC5724862 29228001

[32] Au TC, Nau D. Accident or intention: that is the question (in the Noisy Iterated Prisoner’s Dilemma). In: Proceedings of the fifth international joint conference on Autonomous agents and multiagent systems. ACM; 2006. p. 561–568.

[33] Press WH, Dyson FJ. Iterated Prisoner’s Dilemma contains strategies that dominate any evolutionary opponent. Proceedings of the National Academy of Sciences of the United States of America. 2012;109(26):10409–13. doi: 10.1073/pnas.1206569109 22615375 10.1073/pnas.1206569109 PMC3387070

[34] Li J, Kendall G. A strategy with novel evolutionary features for the iterated prisoner’s dilemma. Evolutionary Computation. 2009;17(2):257–274. doi: 10.1162/evco.2009.17.2.257 19413490 10.1162/evco.2009.17.2.257

[35] LIFL. PRISON; 2008. http://www.lifl.fr/IPD/ipd.frame.html.

[36] project developers TA. Axelrod-Python/Axelrod: v2.2.0; 2016. https://doi.org/10.5281/zenodo.211828.

[37] Ashlock D, Kim EY. The impact of varying resources available to iterated prisoner’s dilemma agents. In: Foundations of Computational Intelligence (FOCI), 2013 IEEE Symposium on. IEEE; 2013. p. 60–67.

[38] Wang Z, Kokubo S, Jusup M, Tanimoto Jun. Universal scaling for the dilemma strength in evolutionary games. Physics of life reviews. 2015;(14):1–30. doi: 10.1016/j.plrev.2015.04.033 10.1016/j.plrev.2015.04.033 25979121

[39] Tanimoto J, Sagara H. Relationship between dilemma occurrence and the existence of a weakly dominant strategy in a two-player symmetric game. BioSystems. 2007 8 31;90(1):105–14. doi: 10.1016/j.biosystems.2006.07.005 17188808 10.1016/j.biosystems.2006.07.005

[40] Hunter JD. Matplotlib: A 2D graphics environment. Computing In Science & Engineering. 2007;9(3):90–95. doi: 10.1109/MCSE.2007.55

[41] McKinney W, et al. Data structures for statistical computing in python. In: Proceedings of the 9th Python in Science Conference. vol. 445. van der Voort S, Millman J; 2010. p. 51–56.

[42] Walt Svd, Colbert SC, Varoquaux G. The NumPy array: a structure for efficient numerical computation. Computing in Science & Engineering. 2011;13(2):22–30. doi: 10.1109/MCSE.2011.37

[43] Li J, Hingston P, Member S, Kendall G. Engineering Design of Strategies for Winning Iterated Prisoner’ s Dilemma Competitions. 2011;3(4):348–360.

[44] Tzafestas E. Toward adaptive cooperative behavior. From Animals to animals: Proceedings of the 6th International Conference on the Simulation of Adaptive Behavior (SAB-2000). 2000;2:334–340.

[45] Mittal S, Deb K. Optimal strategies of the iterated prisoner’s dilemma problem for multiple conflicting objectives. IEEE Transactions on Evolutionary Computation. 2009;13(3):554–565. doi: 10.1109/TEVC.2008.2009459

[46] Hilbe C, Nowak MA, Traulsen A. Adaptive dynamics of extortion and compliance. PloS one. 2013;8(11):e77886 doi: 10.1371/journal.pone.0077886 24223739 10.1371/journal.pone.0077886 PMC3815207

[47] Nachbar JH. Evolution in the finitely repeated prisoner’s dilemma. Journal of Economic Behavior & Organization. 1992;19(3):307–326. doi: 10.1016/0167-2681(92)90040-I

[48] Wu J, Axelrod R. How to cope with noise in the iterated prisoner’s dilemma. Journal of Conflict resolution. 1995;39(1):183–189. doi: 10.1177/0022002795039001008

[49] van den Berg P, Weissing FJ. The importance of mechanisms for the evolution of cooperation In: Proc. R. Soc. B. vol. 282 The Royal Society; 2015 p. 20151382. 10.1098/rspb.2015.1382 PMC4632633 26246554

[50] Arnold E. CoopSim v0.9.9 beta 6; 2015. https://github.com/jecki/CoopSim/.

[51] Frean MR. The prisoner’s dilemma without synchrony. Proceedings of the Royal Society of London B: Biological Sciences. 1994;257(1348):75–79. doi: 10.1098/rspb.1994.0096 10.1098/rspb.1994.0096 8090793

[52] Nowak M, Sigmund K. A strategy of win-stay, lose-shift that outperforms tit-for-tat in the Prisoner’s Dilemma game. Nature. 1993;364(6432):56–58. doi: 10.1038/364056a0 8316296 10.1038/364056a0

[53] Beaufils B, Delahaye JP, Mathieu P. Our meeting with gradual, a good strategy for the iterated prisoner’s dilemma. In: Proceedings of the Fifth International Workshop on the Synthesis and Simulation of Living Systems; 1997. p. 202–209.

[54] Banks JS, Sundaram RK. Repeated games, finite automata, and complexity. Games and Economic Behavior. 1990;2(2):97–117. doi: 10.1016/0899-8256(90)90024-O

[55] Robson AJ. Efficiency in evolutionary games: Darwin, Nash and the secret handshake. Journal of theoretical Biology. 1990;144(3):379–396. doi: 10.1016/S0022-5193(05)80082-7 2395377 10.1016/s0022-5193(05)80082-7

[56] Unknown. www.prisoners-dilemma.com; 2017. http://www.prisoners-dilemma.com/.

[57] Li J, Kendall G, Member S. The effect of memory size on the evolutionary stability of strategies in iterated prisoner’s dilemma. 2014;X(X):1–8.

[58] Ashlock D, Kim EY. Fingerprinting: Visualization and automatic analysis of prisoner’s dilemma strategies. IEEE Transactions on Evolutionary Computation. 2008;12(5):647–659. doi: 10.1109/TEVC.2008.920675

[59] Carvalho AL, Rocha HP, Amaral FT, Guimaraes FG. Iterated Prisoner’s Dilemma-An extended analysis. 2013;.

[60] Adami C, Hintze A. Evolutionary instability of zero-determinant strategies demonstrates that winning is not everything. Nature communications. 2013;4(1):2193 doi: 10.1038/ncomms3193 23903782 10.1038/ncomms3193 PMC3741637

[61] Kraines D, Kraines V. Pavlov and the prisoner’s dilemma. Theory and decision. 1989;26(1):47–79. doi: 10.1007/BF00134056

[62] Kuhn S. Prisoner’s Dilemma In: Zalta EN, editor. The Stanford Encyclopedia of Philosophy. spring 2017 ed. Metaphysics Research Lab, Stanford University; 2017.

